# Cell Wall Proteomics Reveal Phenotypic Adaption of Drug-Resistant *Mycobacterium smegmatis* to Subinhibitory Rifampicin Exposure

**DOI:** 10.3389/fmed.2021.723667

**Published:** 2021-10-05

**Authors:** Alexander D. Giddey, Tariq A. Ganief, Naadir Ganief, Anastasia Koch, Digby F. Warner, Nelson C. Soares, Jonathan M. Blackburn

**Affiliations:** ^1^Division of Chemical and Systems Biology, Department of Integrative Biomedical Sciences, Faculty of Health Sciences, University of Cape Town, Cape Town, South Africa; ^2^South African Medical Research Council/National Health Laboratory Service/University of Cape Town Molecular Mycobacteriology Research Unit, Department of Pathology, Faculty of Health Sciences, University of Cape Town, Cape Town, South Africa; ^3^Institute of Infectious Disease and Molecular Medicine, Faculty of Health Sciences, University of Cape Town, Cape Town, South Africa; ^4^College of Pharmacy, University of Sharjah, Sharjah, United Arab Emirates; ^5^Sharjah Institute for Medical Research, University of Sharjah, Sharjah, United Arab Emirates

**Keywords:** mass spectrometry, proteomics, rifampicin, *Mycobacterium smegmatis*, phenotypic adaption, ABC transporters, sublethal

## Abstract

Despite the availability of effective drug treatment, *Mycobacterium tuberculosis* (Mtb), the causative agent of TB disease, kills ~1. 5 million people annually, and the rising prevalence of drug resistance increasingly threatens to worsen this plight. We previously showed that sublethal exposure to the frontline anti-TB drug, rifampicin, resulted in substantial adaptive remodeling of the proteome of the model organism, *Mycobacterium smegmatis*, in the drug-sensitive mc^2^155 strain [wild type (WT)]. In this study, we investigate whether these responses are conserved in an engineered, isogenic mutant harboring the clinically relevant S531L rifampicin resistance-conferring mutation (SL) and distinguish the responses that are specific to RNA polymerase β subunit- (RpoB-) binding activity of rifampicin from those that are dependent on the presence of rifampicin alone. We verified the drug resistance status of this strain and observed no phenotypic indications of rifampicin-induced stress upon treatment with the same concentration as used in WT (2.5 μg/ml). Thereafter, we used a cell wall-enrichment strategy to focus attention on the cell wall proteome and observed 253 proteins to be dysregulated in SL bacteria in comparison with 716 proteins in WT. We observed that decreased abundance of ATP-binding cassette (ABC) transporters and increased abundance of ribosomal machinery were conserved in the SL strain, whereas the upregulation of transcriptional machinery and the downregulation of numerous two-component systems were not. We conclude that the drug-resistant *M. smegmatis* strain displays some of the same proteomic responses observed in WT and suggest that this evidence supports the hypothesis that rifampicin exercises effects beyond RpoB-interaction alone and that mycobacteria recognise rifampicin as a signaling molecule in an RpoB-independent manner at sublethal doses. Taken together, our data indicates mixed RpoB-independent and RpoB-dependent proteomic remodeling in WT mycobacteria, with evidence for RpoB-independent ABC transporter downregulation, but drug activity-based transcriptional upregulation and two-component system downregulation.

## Introduction

*Mycobacterium tuberculosis* (Mtb) causes TB disease, which accounts for ~1.5 million deaths annually despite the existence of effective drug treatments for ~80 years. Drug resistance continues to grow, threatening global progress in fighting a disease that once claimed the life of one in every five people in eighteenth and nineteenth century Europe (1). Treatment success rates drop from more than 85% in fully drug-susceptible TB cases to ~39% in extensively drug-resistant TB cases (XDR-TB) (2), rising again to ~90% in XDR-TB cases in the ongoing Nix-TB clinical trial utilising a novel drug regimen of pretomanid (PA-824), bedaquiline, and linezolid—three of the four new TB drugs approved for use since rifampicin in 1968, with pretomanid only approved as recently as 2019 (2–5). The slow pace of discovery of new anti-TB drugs underscores the risk that, unless the ongoing acquisition of drug resistance can be prevented or slowed, we may yet face catastrophic failure of TB chemotherapy generally, with survival rates returning to those seen in previous centuries.

Mycobacteria develop antibiotic resistance primarily through point mutations rather than by horizontal gene transfer (6). Although their base mutation rate is low, they possess means by which this can be increased. Drugs, such as the fluoroquinolones, can activate damage-induced mutagenesis pathways by which error-prone replication machinery, such as DnaE2, can enable both survival of the bacterium through translesion synthesis, and increased mutation frequency—allowing for the evolution of drug resistance (7, 8). Another mechanism by which bacteria can acquire resistance is through initial phenotypic adaption to sublethal drug exposure. Bacteria continue to survive and replicate at doses below that of the minimum inhibitory concentration (MIC) of the drug, but with a gentle selective pressure that favours the acquisition of phenotypic resistance, followed by the evolution of resistant mutants with low fitness costs (9). In contrast to supra-MIC resistance selection that assumes preexisting mutants with a high level of resistance, this mode of resistance selection allows for the *de novo* development of drug resistance. Additionally, while it might be naively assumed that antibiotics applied in the treatment of disease are foreign to the target microbe and exercise their activity using the designated primary mechanism with few other ramifications, in reality, a number of antibiotics are natural products, which were first discovered in microorganisms (10, 11) and at low concentrations can behave as cell signaling molecules, triggering significant changes in gene transcription and often in gene subsets capable of counteracting the applied antibiotic (12, 13). In this regard, it is important to note that mycobacteria encode numerous intrinsic resistance factors, which can be readily dysregulated when needed. These include drug inactivating enzymes such as the broadly active BlaC β-lactamase of Mtb (14); transporters such as MmpL7 capable of performing double duty as drug efflux pumps (15); or general impermeability to drug, mediated by the unusually thick, lipid-rich, hydrophobic mycobacterial cell wall (16). Unlike with acquired resistance, these intrinsic resistance factors are found generally throughout a population of bacilli and do not require a selection process. Such resistance can thus be adaptive and phenotypic rather than evolutionary, in that bacteria can regulate such responses within the existing genetic framework. Slow growing and generally slow mutating organisms such as Mtb, are well-served by adaptive programs for resistance, as epitomised by the mycobacterial DosR-regulated dormancy response pathway, through which Mtb is able to increase survival both against various drugs and the immune system (17, 18).

The natural product, rifampicin, is a first-line antitubercular drug and, along with isoniazid, pyrazinamide, and ethambutol, forms part of the current 6-month short-course TB drug regimen. The primary mechanism of action of rifampicin involves its binding to the RNA polymerase β subunit (RpoB), thereby physically inhibiting RNA transcript elongation (19). Numerous point mutations exist which confer rifampicin resistance in mycobacteria. The most frequent and clinically relevant of these, in Mtb, is the S531L mutation which can account for ~43% of all drug-resistant isolates (20, 21). This mutation occurs in the 81 bp rifampicin resistance determining region (RRDR) of RpoB (22). By convention, these mutation codons are numbered for the related codon in mutant *Escherichia coli* where the RRDR was first discovered: the S531L mutation in *E. coli* RpoB is matched to S456L of the Mtb sequence (Uniprot entry P9WGY9) and S447L in *Mycobacterium smegmatis* (Uniprot entry P60281). The S531L mutation in the RRDR results in the larger amino acid leucine filling the rifampicin binding pocket, hindering binding of the drug, and thus giving rise to high-level drug resistance (6, 23). This makes the S531L rifampicin-resistant mycobacterial mutant highly complementary to wild type (WT) in the study of sublethal drug effects as the development of such resistance protects bacteria from the primary action of the drug but is not expected to prevent its intracellular accumulation. This S531L mutant thus provides an excellent means to test whether bacterial responses to sublethal rifampicin are consequent to the primary mode of drug action or whether they are in response to secondary stresses or other upstream drug interactions such as *via* signaling mechanisms.

We previously reported that a subinhibitory dose of rifampicin in the treatment of drug-susceptible (WT) *M. smegmatis* mc^2^155 resulted in increased abundance of both transcriptional and translational cellular machinery, decreased abundance of numerous ABC transporters, and eventual upregulation of the *M. smegmatis*-specific rifampicin inactivating enzyme, ADP-ribosyl transferase (arr) (24, 25). We also observed dysregulation in iron metabolism, various cell wall synthesis enzymes, virulence factors, and two-component system proteins. Following such dysregulation, bacteria were able to acquire phenotypic resistance, thereby overcoming growth inhibition and resuming growth comparable to untreated controls. In this study, by treatment of the isogenic drug-resistant S531L mutant (SL) with the same sublethal rifampicin concentration, we distinguish the proteomic responses that are WT-specific from those that are conserved in SL bacteria, thereby differentiating responses to the presence of rifampicin—and potentially its recognition as a signaling molecule—from those responses which are dependent on the primary mechanism of drug action. By so doing, we both validate previously observed proteomic results and generate new hypotheses for future investigations.

## Materials and Methods

### Strain Information

*Mycobacterium smegmatis* drug-resistant mutant strain (SL) was generated from *M. smegmatis* mc^2^155 by the engineered mutation of codon 531 in the RRDR from serine to leucine (S531L) and was validated by sequencing. The complementary or reversed double-mutant (i.e., WT -> S531L -> L531S) was also generated, but the data on this strain are not reported in this study, except to note that MIC and growth characteristics of the reverse complement reverted to those of the WT, providing high confidence in the specificity of S531L mutation.

### Determination of Sublethal Rifampicin Concentration

The MIC of rifampicin for the SL strain was determined using a micro-broth dilution method. Briefly, rifampicin was solubilised in dimethyl sulfoxide (DMSO) to 10 mg/ml and diluted to 8,000 μg/ml as the initial rifampicin concentration before serial 2-fold dilution in oleic acid-albumin-dextrose-catalase- (OADC-) enriched 7H9 media, with either Tween-20 (0.1%) or Tween-80 (0.05%), followed by the addition of mid-growth *M. smegmatis* diluted to the equivalent of A_600nm_ 0.006. No drug was added to the final row, and no bacteria were added to the first as positive and negative controls, respectively. Plates were sealed in zip lock bags and incubated at 37°C for 3–4 days before visual inspection for growth.

For the determination of the sublethal rifampicin drug concentration by treatment curve, duplicate flasks of 75 ml liquid cultures in Tween-20 (0.1%, see subsection Growth Conditions and Harvesting) in log phase growth (A_600nm_ 1.2) were treated with rifampicin solubilised in DMSO to a final concentration of 2.5 μg/ml, or with DMSO only as a control. Cell density, and hence bacterial growth, was inferred from A_600nm_ readings measured every 1.5–2.5 h from 2 h prior to treatment until 7 h post-treatment and then again 52 h post-treatment using a Varian Cary 50 UV-visible spectrophotometer.

### Growth Conditions and Harvesting

*Mycobacterium smegmatis* SL strain was grown as done previously, except that volumes were scaled to 75 ml (24, 25). Briefly, bacteria were grown in 7H9 broth supplemented with 0.1% Tween-20, 0.2% glycerol, and 10% OADC with four flasks per treatment group, yielding a total of 8 samples. On reaching an optical density (A_600nm_) of 1.2, the cultures were treated with either 2.5 μg/ml rifampicin in DMSO (treated) or with DMSO only (control). The bacterial cultures were harvested at 255 min post-treatment by the transferal of each culture to two 50 ml centrifuge tubes and centrifugation of 3,000 relative centrifugal force (RCF) at 8°C for 15 min. Pellets were washed twice by resuspension in phosphate buffered saline (PBS), snap frozen *via* liquid nitrogen, and stored at −80°C before lysis and cell wall enrichment.

### Cell Wall Protein Preparation

Washed cell pellets were resuspended in lysis buffer [PBS pH 7.4, protease inhibitors (Roche, Basel, Switzerland)] and lysed by sonication for six rounds of 30 s each with 1 min cooling in an ice/water slurry. Cell debris (see below) were pelleted by centrifugation at 8,000 RCF for 10 min and the lysates filtered through a 0.2 μm filter before pelleting the cell wall fraction by centrifugation at 22,000 RCF for 30 min at 4°C. The supernatant containing the cytosolic fraction was precipitated with chloroform/methanol (26) and the protein pellet was solubilised in UA buffer (8 M urea, 0.1 M Tris, and pH 8.5). The cell wall pellet was washed twice with PBS and resuspended in 0.1% Triton X100, 0.05% Tween-20, and 0.2% 3-[(3-cholamidopropyl)dimethylammonio]-1-propanesulfonate (CHAPS) (25). Insoluble aggregates were removed by centrifugation at 22,000 RCF. See Supplementary Figure 1 for a graphical schema adapted from Hermann et al. (27).

### Cell Debris Preparation

The residual cell debris pellets were washed once with PBS, resuspended in 0.1 M Tris, 2% sodium dodecyl sulfate (SDS), and 100 mM dithiothreitol (DTT), pH 8.5, and boiled at 95°C for 1 h. The samples were cooled to room temperature on ice and mixed with urea to a final concentration of 6 M by shaking for 30 min at room temperature.

### Filter-Aided Sample Preparation

All proteome fractions—the cytosolic, the cell wall, and the cell debris fractions—were precipitated *via* chloroform/methanol and solubilised in UA buffer before using filter-aided sample preparation (FASP) for digestion (28). Centrifugation steps were performed at 14,000 RCF for 15 min. 50 μg of each sample was added into separate Ultracel 30,000 molecular weight cutoff (MWCO) centrifugal units (Amicon Ultra, Merck, Darmstadt, Germany) and washed three times with UA buffer. Proteins were reduced by the addition of 100 mM DTT for 30 min at room temperature, and excess DTT was removed by centrifugation. Reduced cysteines were alkylated by the addition of 0.05 M iodoacetamide for 20 min in the dark at room temperature. The alkylating reagent was removed by three rounds of buffer exchange with UA buffer followed by three rounds of buffer exchange into ABC buffer (0.05 M ammonium bicarbonate pH 8.0, 20 mM CaCl_2_).

Sequence-grade trypsin (New England Biolabs, Ipswich, MA, USA) was added at an enzyme to protein ratio of 1:50 and digestion proceeded for 18 h at 37°C in a humidified chamber. Tryptic peptides were eluted by three rounds of buffer exchange with ABC buffer and desalted using homemade STAGE tips containing Empore Octadecyl C18 solid-phase extraction disks (Supelco, Bellefonte, PA, USA). C18 disks were activated with three rounds of washing with solvent A (80% acetonitrile and 0.1% formic acid) and equilibrated with three rounds of washing with solvent B (2% acetonitrile and 0.1% formic acid). 10 μg of tryptic peptides was loaded on the C18 disc, centrifuged at 4,000 RCF for 1 min, and washed three times with solvent B. Peptides were then eluted three times with solvent C (60% acetonitrile and 0.1% formic acid) into glass capillary tubes. The eluted peptides were dried in a vacuum and resuspended with solvent A (2% acetonitrile and 0.1% formic acid) at a concentration of 200 ng/μl.

### Liquid Chromatography-Tandem Mass Spectrometry Analysis

Chromatographic separation was achieved using a 100 μm ID, 20 mm pre-column connected to a 75 μm, 300 mm analytical column packed with C18 Luna beads (5 μm diameter, 100 Å pore size; Phenomenex 04A-5452) connected to an Ultimate 3500 RS nano UPLC system (Dionex, Sunnyvale, CA, USA). Approximately 600 ng of desalted peptides per sample were loaded onto the column with a starting mobile phase of 2% acetonitrile (ACN), 0.1% formic acid, and separated at a constant flow rate of 400 nl/min over a 70 min gradient as follows: 10 min at 2% ACN, increased to 6% ACN over 2 min, to 40% ACN over 70 min, and to 80% ACN over 5 min, and held at 80% for 15 min as a column wash.

Mass spectra were collected on a Q Exactive mass spectrometer (Thermo Fisher Scientific, Waltham, MA, USA) operating in positive mode, with data-dependent acquisition and a top-10 method. Intensity threshold for MS^2^ ion selection was 1.3e4 with charge exclusion of *z* = 1 and *z* > 5. Peptides were ionised by electrospray ionisation, and MS spectra were acquired at a resolution of 70,000 for MS^1^ and 17,500 for MS^2^. Automated gain control (AGC) target was set to 3e6 with a maximum integration time of 250 ms (MS^1^) and 1e5 with a maximum integration time of 80 ms (MS^2^). MS^1^ scan range was 300–1,750 Da, and peptide fragmentation was performed using higher-energy collision dissociation (HCD) and setting the energy to 28 Normalised Collision Energy (NCE) and the product ion scan range to 200–2,000 Da.

Subsequently, the data from these samples were reacquired. MS acquisition parameters were unchanged while chromatographic conditions differed in that the 70 min gradient was from 6% ACN to 23%, rather than to 40%. The results obtained from the first dataset are presented, except where the consideration of the combined datasets for greater statistical power is explicitly mentioned.

### Bioinformatic Analysis

RAW data files were processed with Maxquant (29) version 1.5.3.12 for protein and peptide identification using the Andromeda (30) search engine and the Uniprot proteome for *M. smegmatis*. Default parameter settings were used for the MS/MS database search, with the selection of carbamidomethylation of cysteine residues and acetylation of the protein N-terminus as fixed modifications. As per the default settings, trypsin/P was used as the *in silico* protease, and intensity-based absolute quantification (iBAQ) and *match between runs* were enabled. Protein identifications were required to pass a false discovery rate (FDR) of 1% with one or more unique peptides for inclusion in further analysis.

The *proteinGroups* file was subsequently analysed in R using a custom script to remove contaminants and reverse hits. Data iBAQ values were median normalised, and an exploratory data analysis was performed by principal component analysis (PCA) plots and hierarchical clustering. The cell wall fraction of one sample (sample 6R) was identified to be an outlier and removed from further analysis (see Results subsection Data Quality and Exploratory Analysis). The significance of altered abundance was determined by means of the Student's *t*-test with *p* < 0.05, without multiple-testing correction, for all proteins with at least three non-zero values in each treatment group. Those proteins with abundance dysregulated by more than twice the SD of fold changes across the proteome for that compartment (cell debris, cell wall, or cytosolic) were considered to be strongly dysregulated. Lists of dysregulated proteins were subsequently tested for gene ontology (GO) term and Kyoto encyclopedia of genes and genomes (KEGG) pathway (31–33) enrichment using the *STRINGdb* package (34, 35).

In this paper, comparisons to the results in our former longitudinal study [see (24)] are made after reanalysing the previous data so as to consider iBAQ-based results of that study to avoid any possible bias in comparing LFQ to iBAQ-based quantitation. Where reference is made to the combined SL datasets, batch correction was performed using the ComBat algorithm (36).

## Results

### Determination of Sublethal Rifampicin Concentration (SL)

**Figure 1:**
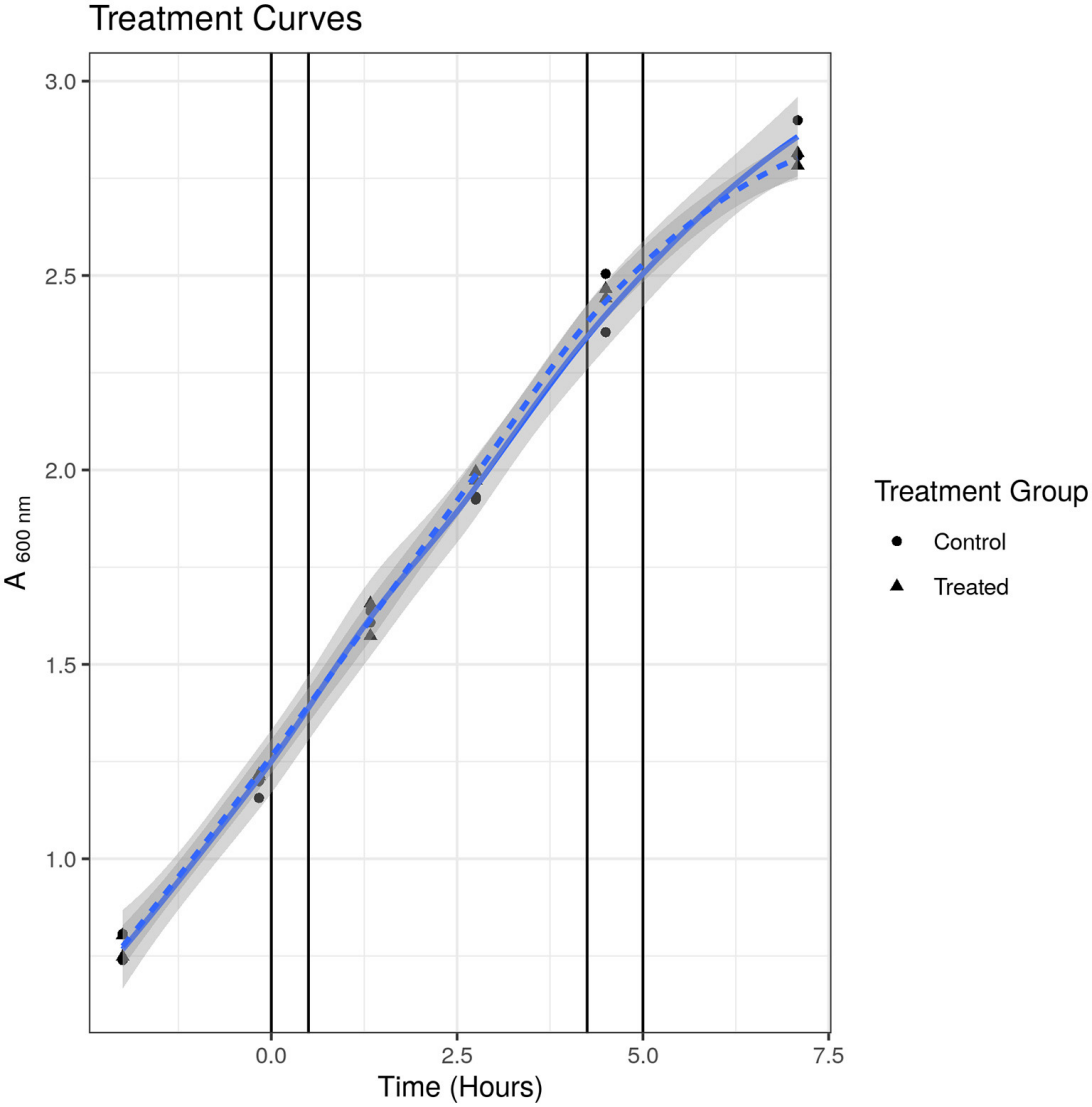
Treatment curve for SL mutant. Treated indicates samples treated with 2.5 μg/ml. Control indicates samples treated with solvent [dimethyl sulfoxide (DMSO)] only. Points indicate individual replicate values. Lines represent fitted loess models. There was no observable difference in growth at any time point measured.

To check that the S531L mutant was indeed resistant to rifampicin, we measured the MIC using the micro-broth dilution method and treatment curves. We determined the MIC of rifampicin for SL bacteria to be 15.63 μg/ml in Tween-20-containing media and 250 μg/ml in Tween 80-containing media, as compared to 0.63 and 2.5 μg/ml, respectively, for susceptible WT bacteria, thus confirming the resistant phenotype. We tested the growth phenotype displayed in Tween-20 containing media when treated at the same sublethal concentration of 2.5 μg/ml that was used in our previous published studies (24, 25) and, as it can be seen in Figure 1, there was no observable impediment to growth at this concentration up to 7 h and at 52 h post-treatment (final data point not shown). We were thus satisfied that there was no phenotypic evidence of drug-induced stress for drug-resistant bacteria under these experimental conditions.

### Data Quality and Exploratory Analysis

**Figure 2:**
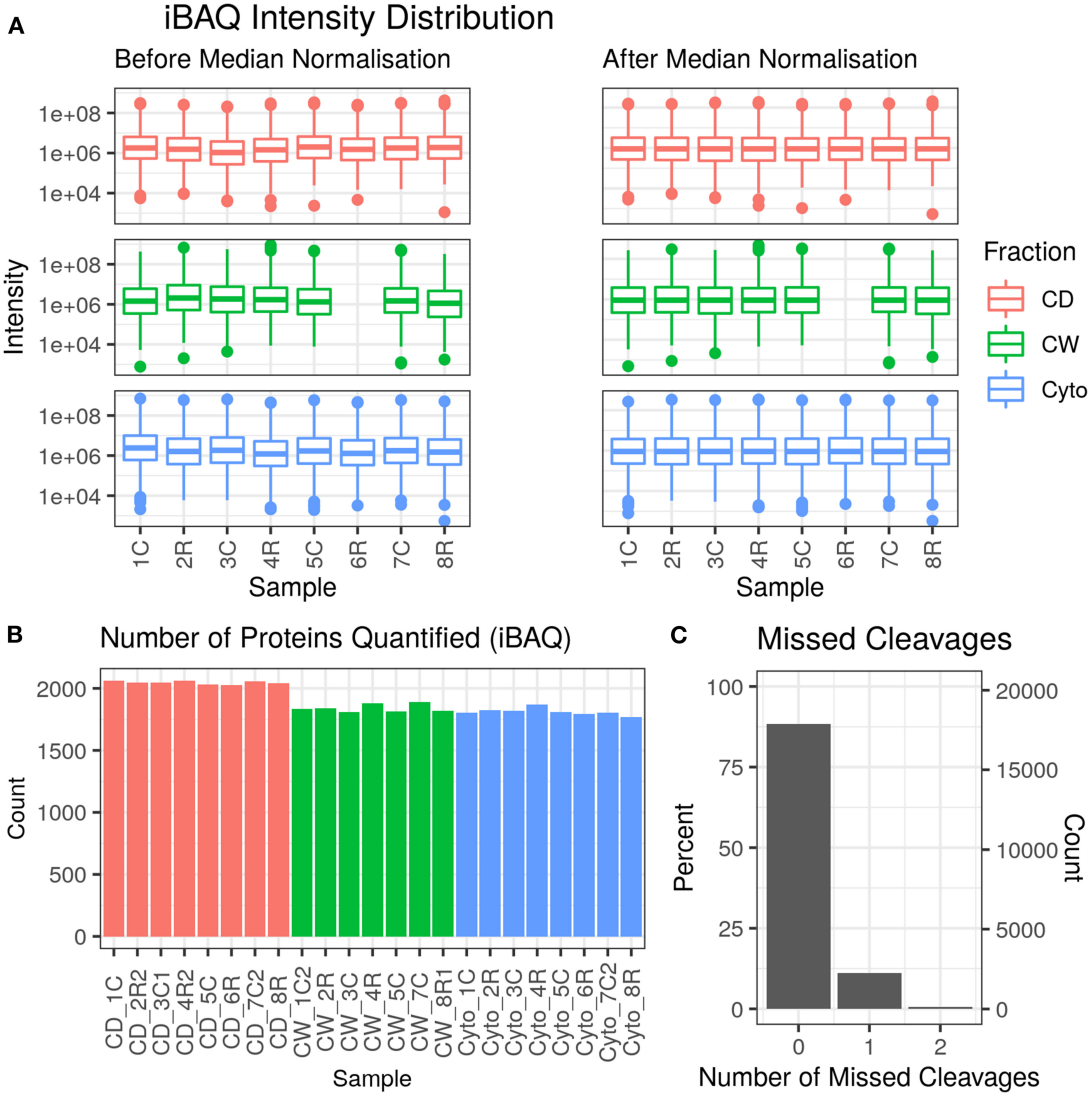
Data quality for primary dataset. **(A)** Distribution of intensity-based absolute quantification (iBAQ) protein quantifications by sample both before and after median normalisation. **(B)** Number of quantified proteins by sample. Colour scheme for **(A,B)** is red for cell debris, green for cell wall, and blue for cytosolic fractions. **(C)** Frequency of number of missed trypsin cleavage sites in identified peptides revealing nearly 90% of the identified peptides showed zero miscleavages.

Across 23 samples, we observed a total of 2,283 proteins and 20,237 peptides, with medians of 1,880 proteins and 8,134 peptides per sample. Proteins were identified with a median of six unique peptides per protein group with nearly all (99%) protein groups consisting of one protein each. Figure 2 shows the protein abundance distributions by sample before and after median normalisation, having excluded zero values, for the primary dataset that is analysed and discussed in this paper. Supplementary Figure 2 shows the same for the second, supplementary dataset where we observed 2,560 proteins, with 2,201 in common with the first (96.4% overlap).

**Figure 3:**
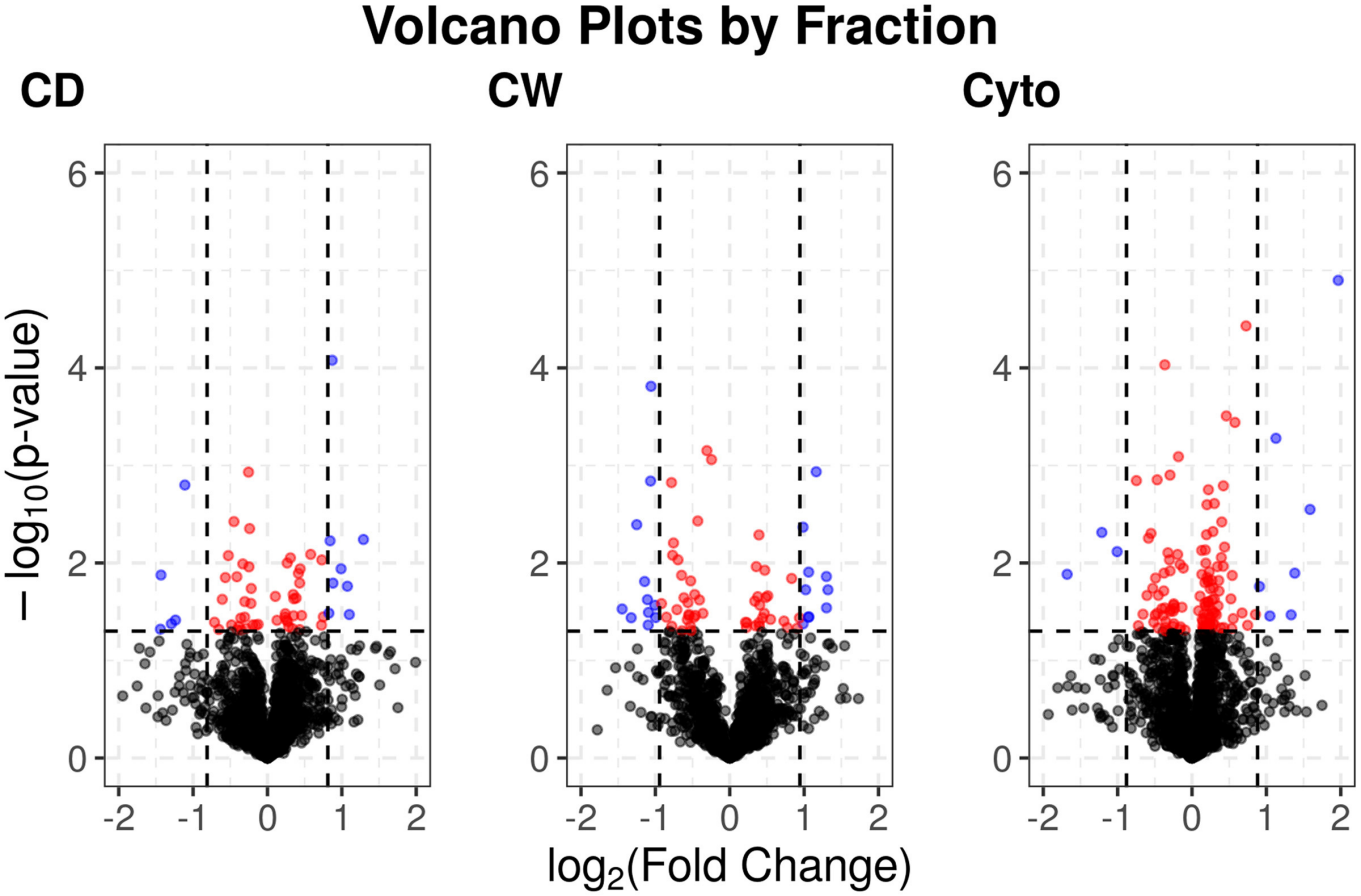
Volcano plots for SL proteins by cellular fraction. Proteins dysregulated upon rifampicin treatment. Red and blue dots indicate proteins with *p* < 0.05. Blue dots have log_2_ fold changes >2 times the SD for fold changes in that cellular fraction.

Supplementary Figures 3, 4 show PCA plots for those SL samples of the primary dataset before and after the identification of the cell wall fraction for sample 6R as an outlier and its exclusion from further analysis. The processing of the remaining samples showed excellent reproducibility with median Pearson correlation scores between biological replicates for cytosolic, cell debris, and cell wall fraction control samples of 99, 98, and 97%, respectively. In total, we observed 253 dysregulated proteins in this study (73 in the cell wall fraction, 63 in the cellular debris fraction, and 128 in the cytosolic fraction). Table 1 presents the 10 most strongly dysregulated proteins (by fold change) in each fraction, and Figure 3 shows volcano plots indicating both the magnitude and significance of protein dysregulation in each cellular fraction.

**Table 1:** Top-10 most dysregulated SL proteins by cellular fraction.

**Fraction**	**Uniprot ID**	**Gene name**	**Protein description**	***p*-value**	**log_**2**_ (FC)**
CD	A0QQB8	MSMEG_0699	Conserved hypothetical proline rich protein	<0.001	2.643
CD	A0QX92	MSMEG_3216	Peroxiredoxin Q	0.038	−2.347
CD	A0QVH8	Rip1	Zinc metalloprotease Rip1	<0.001	2.314
CD	A0R0H2	MSMEG_4385	ABC transporter oligopeptide binding protein	0.048	−1.439
CD	A0QVI7	CobQ	Cobyric acid synthase	0.013	−1.432
CD	A0QNH6	SaeC	Putative ESX-1 scaffolding and assembly protein SaeC	0.042	−1.293
CD	A0QRE7	MSMEG_1090	Amidase	0.006	1.291
CD	A0R3Y8	MSMEG_5642	Acetyl-coenzyme a carboxylase carboxyl transferase	0.039	−1.237
CD	A0R3I6	MSMEG_5486	Peptidase S1 and S6, chymotrypsin/Hap	0.002	−1.111
CD	A0R3U0	MSMEG_5593	Pyruvate dehydrogenase	0.034	1.100
CW	A0QV41	MSMEG_2439	LppW protein	0.013	−2.846
CW	A0QVH8	Rip1	Zinc metalloprotease Rip1	0.009	2.549
CW	A0QQB8	MSMEG_0699	Conserved hypothetical proline rich protein	<0.001	2.361
CW	A0QZI2	MSMEG_4034	NAD dependent epimerase/dehydratase family protein	0.001	−2.030
CW	A0QNR0	MSMEG_0132	Uncharacterised protein	0.03	−1.447
CW	A0QYD4	MSMEG_3619	Short chain dehydrogenase	0.036	−1.325
CW	A0QRP9	MSMEG_1195	Uncharacterised protein	0.019	1.320
CW	A0R1W4	MSMEG_4896	Non-ribosomal peptide synthetase	0.029	1.301
CW	A0QVI7	CobQ	Cobyric acid synthase	0.014	1.300
CW	A0QRG7	MSMEG_1111	Uncharacterised protein	0.004	−1.251
Cyto	A0QVD8	MSMEG_2539	Thiopurine S-methyltransferase (Tpmt) superfamily protein	<0.001	6.348
Cyto	A0R5E2	MSMEG_6160	ATP-dependent rna helicase, dead/deah box family protein	0.025	−3.802
Cyto	A0R037	MSMEG_4248	1-acylglycerol-3-phosphate O-acyltransferase	0.007	2.053
Cyto	A0QUL6	MSMEG_2252	Flavin-type hydroxylase	<0.001	1.966
Cyto	A0QUX7	IlvH	Acetolactate synthase small subunit	0.013	−1.679
Cyto	A0QZ86	MSMEG_3935	Uncharacterised protein	0.003	1.587
Cyto	A0QV60	MSMEG_2458	Uncharacterised protein	0.013	1.382
Cyto	A0R4L0	MSMEG_5870	Sensor histidine kinase PhoR	0.034	1.333
Cyto	A0R129	LppH	Putative conserved lipoprotein lpph	0.005	−1.213
Cyto	A0QVD7	MSMEG_2538	MarR-family protein transcriptional regulator	0.001	1.128

*Ordered by the absolute magnitude of fold change*.

### Enrichment Efficacy

**Figure 4:**
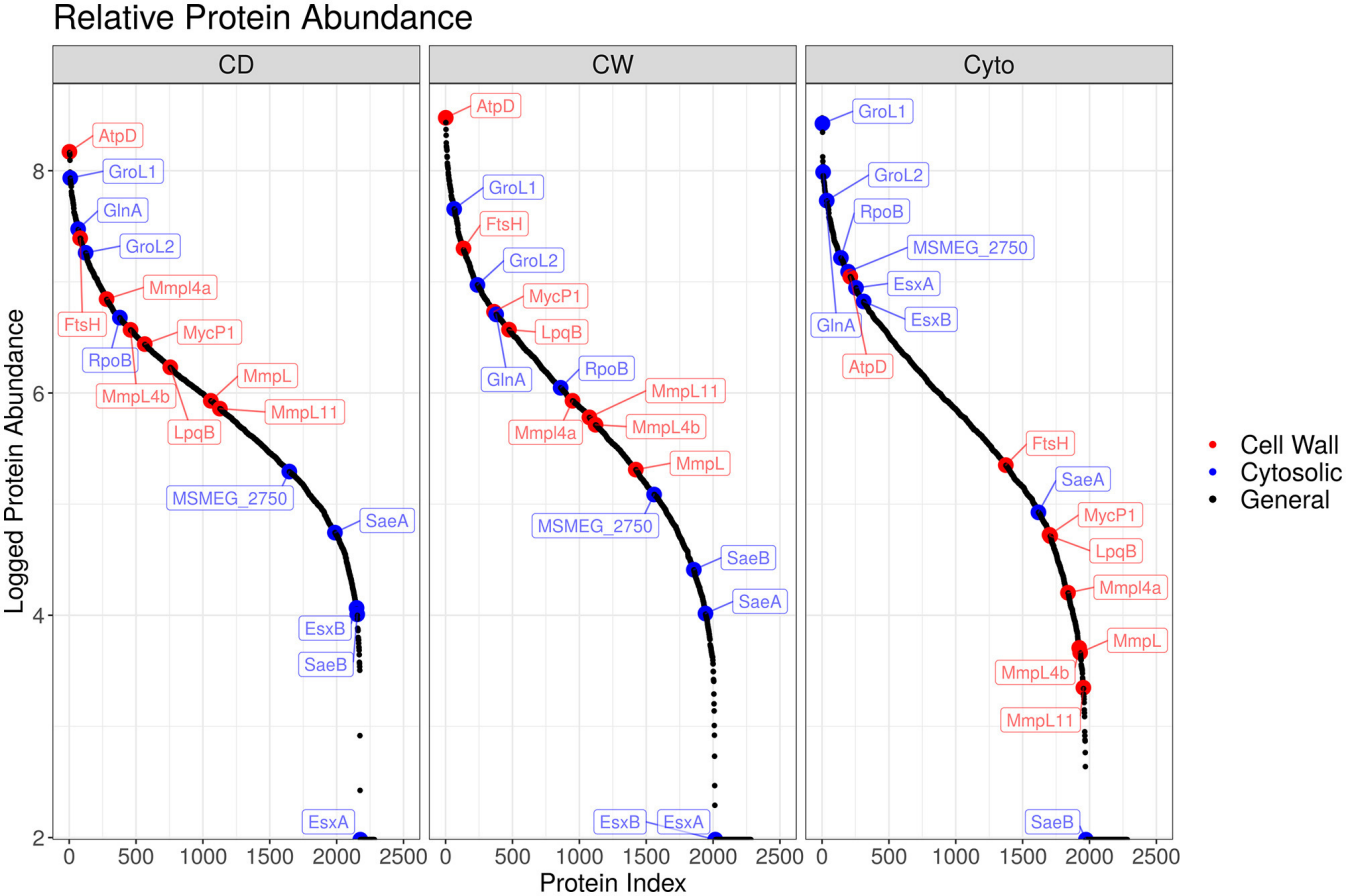
Enrichment efficacy for SL bacteria. Ordered plot of proteins and their iBAQ abundance. Red points indicate known cell wall markers and blue cytosolic. A clear enrichment in the relative abundance of cell wall proteins within the cell wall and cell debris fractions over that observed in the cytosolic fraction is evident.

Overall, we were able to quantitatively compare 432 membrane-annotated proteins, of which 399 were annotated using the QuickGO annotation search (37) as integral membrane components. Figure 4 shows the relative enrichment of cell wall-associated proteins, such as AtpD, MmpL, and LpqB, in the cell wall and cell debris fractions compared to that in the cytosolic fraction, confirming that our enrichment strategy was successful.

### Responses Conserved Across Drug Resistance Status

The rationale for examining the response of drug-resistant *M. smegmatis* to sublethal drug dosage lies in determining which responses observed in drug-susceptible strains are carried through to isogenic drug-resistant strains, where the antibiotic activity of the drug is absent due to the point mutation on the RpoB subunit. We observed that some pathways, but not all, that were previously reported to be dysregulated in WT strains in the presence of sublethal rifampicin were also dysregulated in this drug-resistant strain.

#### ABC Transporters Are Decreased in SL Mutants

The most clearly identifiable of those responses observed in WT which were conserved in this mutant strain was the downregulation of ABC transporters. Here, we observed 11 of 12 dysregulated ABC transporter proteins to be decreased in response to rifampicin treatment, which included mammalian cell entry (MCE) protein Ms5897. When we consider both SL datasets together, the evidence is even more convincing with all (22 of 22) dysregulated members of this pathway decreased in abundance. This is compared with 14 of 22 in our previous longitudinal dataset (4 of 9 at this time point) (24) and 22 of 29 in our drug-susceptible, cell wall-enriched data (25). Altogether, across all three datasets, we observed 34 of 46 dysregulated ABC transporters to be downregulated. The dysregulation of 16 of these ABC transporters was observed at least twice in separate experiments, with all those that were downregulated in our previous longitudinal study (24) also downregulated in our subsequent drug-susceptible, cell wall-enriched study (25). Here, we observed that 6 of the 11 ABC transporters downregulated in the drug-resistant strain are common with those downregulated in the WT.

**Figure 5:**
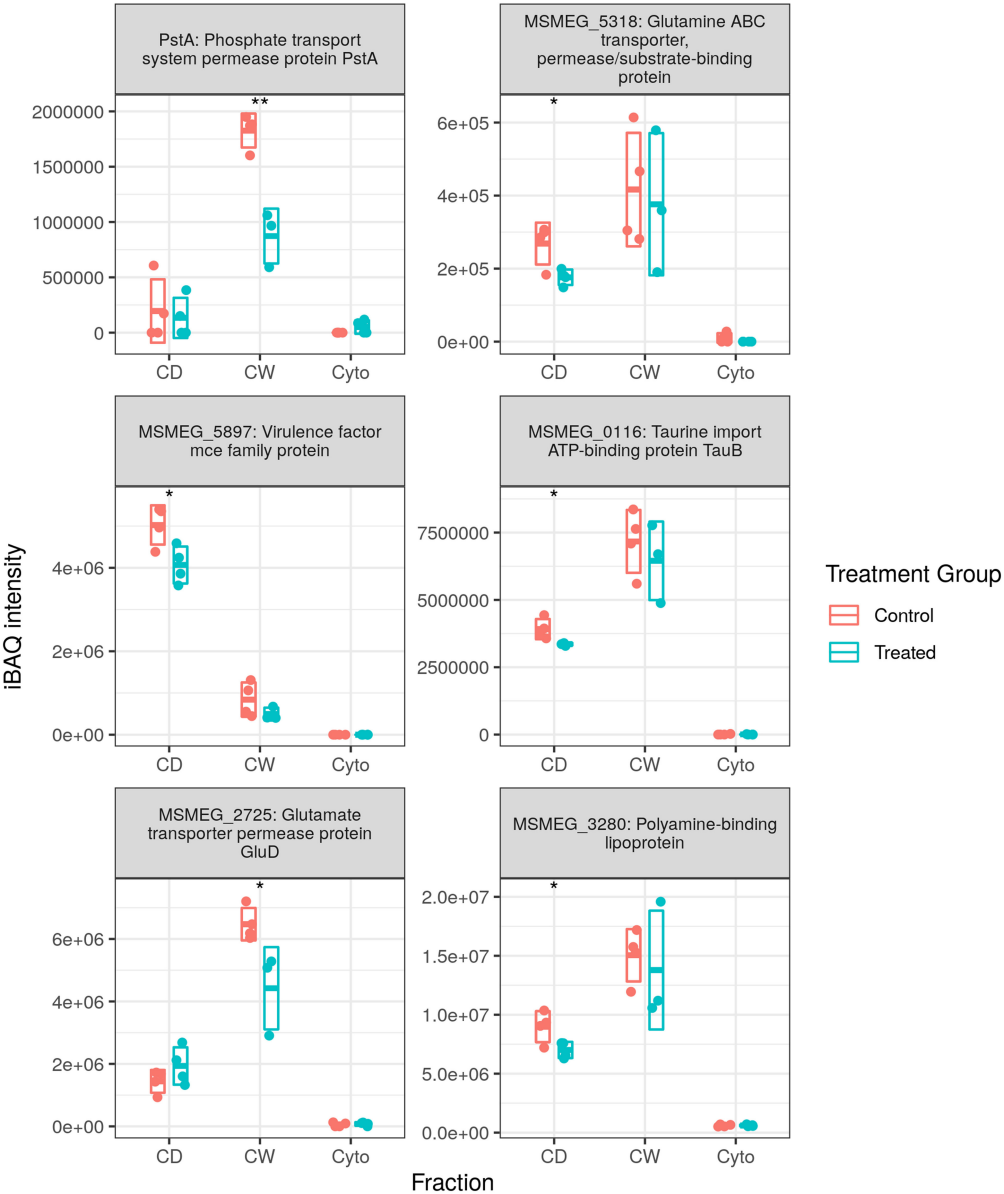
Dysregulated ATP-binding cassette (ABC) transporter proteins. Examples of protein abundance distributions for several ABC transporter proteins across the different fractions in SL mycobacteria. Points indicate individual replicate values and the bars the mean plus/minus SD. Blue indicates control and red rifampicin-treated samples. * and ** indicate *p* < 0.05 and *p* < 0.01 respectively.

Figure 5 shows the measured abundance profiles for several dysregulated ABC proteins. Glutamine transporting Ms5318 and taurine transporting Ms0116 were observed to be downregulated in both WT and SL samples. Glutamate permease GluD was also observed to be among those downregulated in SL samples. Upon further examination, the ABC transporters downregulated in previously generated WT data included taurine, glutamine, glutamate, and unspecified amino acid importers, as well as a nitrate importer and several sulfate importers—including CysA and CysT, involved in cysteine biosynthesis—and methionine importer, MetN. With this in mind, we combined the protein lists and iBAQ quantitation from our two separate MS acquisition datasets for the SL samples, the increased technical replicate numbers providing a greater statistical power to examine ABC transporter dysregulation in SL bacteria. Accordingly, in the combined dataset, we observed the downregulation of the same MetN, sulfonate (Ms0550), and additional glutamine (Ms6307) importers as in the WT. We also additionally observed the downregulation of Ms3247, a branched chain amino acid importer. Between both WT and SL data, we also observed the downregulation of all gene products from Ms2725 through Ms2728—all of which are involved in glutamate import. Thus, our data show that a significant portion of the downregulation of ABC transporters specifically targets amino acid uptake, and that this is true for drug-resistant as well as drug-susceptible mycobacteria, implying a mechanism that is independent of direct transcriptional effects resulting from rifampicin binding to RpoB.

#### Dysregulation of Translational but Not Transcriptional Machinery Is Conserved in SL Bacteria

We previously observed substantial upregulation in both transcriptional and translational machinery with RNA polymerase (RNAP) subunits A, B, and C all increased over time in our longitudinal cell wall-enrichment data in multiple cellular fractions (24, 25). Twelve and thirteen ribosomal proteins, respectively, were previously observed to be dysregulated in our original cell wall enriched (11 increased) and longitudinal (13 increased) WT datasets at this time point. In contrast, here we observed an increase in only one RNAP subunit (RpoA) and in only the cytosolic fraction. We also observed two ribosomal proteins that were upregulated with an equal number downregulated (see Supplementary Figure 5), and ribosomal silencing factor RsfS was also strongly downregulated, providing inconclusive evidence for the upregulation of either transcriptional or translational machinery. In combination with the data from the second set of acquisition runs, we see that an additional three ribosomal proteins are significantly upregulated, for a total of five of seven, while RpoA falls out of significance. Thus, it appears that our data presented here indicates that the upregulation of translational machinery, but not transcriptional, is at least partially conserved in the response of drug-resistant strains to rifampicin.

**Figure 6:**
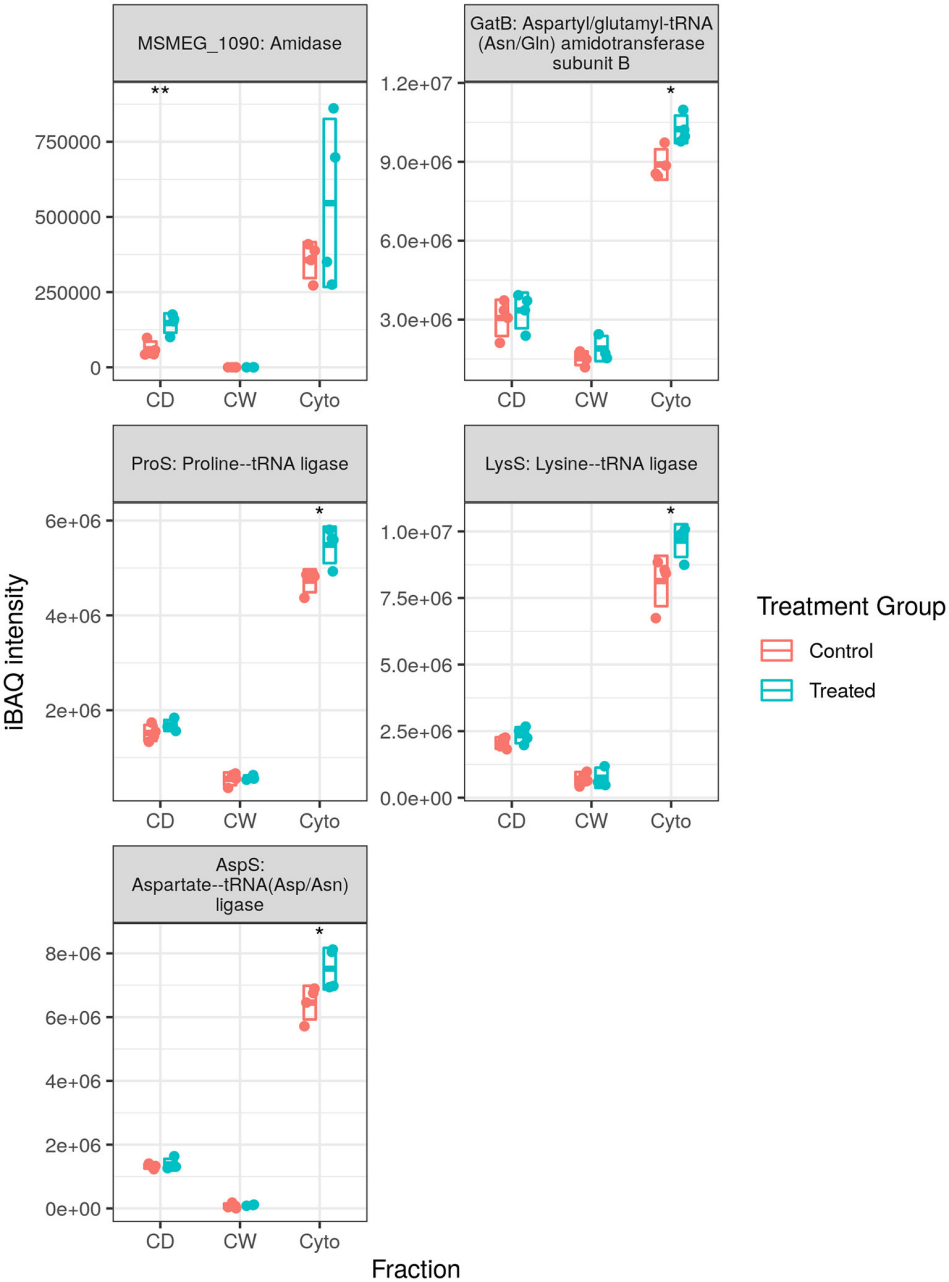
Expression profiles for transfer RNA (tRNA) synthesis enzymes. The trend of generally increased aminoacyl-tRNA synthesis enzyme abundance was conserved in SL samples, albeit with fewer enzymes than that observed in wild-type (WT) samples. Points indicate individual replicate values measured, bars indicate mean expression with error bars indicating SD. Control samples shown in blue and rifampicin treated in red. * and ** indicate *p* < 0.05 and *p* < 0.01 respectively.

We observed 5 transfer RNA (tRNA) synthesis enzymes upregulated in SL bacteria with none downregulated (see Figure 6), whereas in WT bacteria 8 (one at this time point) and 10 tRNA synthesis enzymes were observed to be upregulated in our longitudinal and cell wall-enriched data, respectively. Given the number of amino acid ABC import proteins, and ABC transporters in general, downregulated upon rifampicin exposure, it may be that a reduced concentration of amino acids requires greater concentrations of aminoacyl-tRNA synthesis enzymes to maintain a steady rate of synthesis for translation. Alternatively, this may represent a complementary, specific response to rifampicin, providing an additional mechanism for enhancing translational efficiency given the normal activity of rifampicin in hindering transcription. That this response is observed in SL mycobacteria, comparatively immune to RpoB binding and RNAP interference of rifampicin, would seem to corroborate the finding that ribosomal machinery is upregulated in response to rifampicin exposure regardless of whether or not the bacteria register cellular stress. It is also possible that aminoacyl-tRNA synthesis dysregulation may serve to favour some tRNAs over others as a means of adaptation as proposed by Javid et al. (38). However, given that we observe increased tRNA synthesis enzymes related to 10 of 20 amino acids in the WT, it is not clear if this would prove to be the case.

#### Dysregulation of Porphyrin Synthesis Pathway and Iron Metabolism Is Partially Conserved

We observed substantial dysregulation in the KEGG “porphyrin and chlorophyll metabolism” pathway in SL bacteria. In previous studies on WT, we observed increased porphyrin synthesis and downregulation of flux toward vitamin B12 synthesis. In the subsequent, fractionated study on WT, the observed evidence indicated a mixed dysregulation with respect to vitamin B12 flux, rather than predominant downregulation. Here, in the drug-resistant mutant (see Supplementary Figure 6), we again saw mixed dysregulation similar to that seen in the WT cell wall-enrichment study, with the majority of dysregulated enzymes upregulated but with cytochrome aa3 controlling protein—Ms3117, responsible for converting haem O to haem A—downregulated, as observed previously in WT (24). Cobyric acid synthase, CobQ (Ms2588), appeared to translocate from the cellular debris fraction to the cell wall fraction in this data as this protein is strongly downregulated in the cellular debris fraction and strongly upregulated in the cell wall fraction.

In WT studies, “iron-sulfur cluster” was an enriched GO term in those proteins seen to be dysregulated. The iron-sulfur cluster is a primitive prosthetic group involved in multiple essential processes. Some examples of important proteins and complexes requiring this prosthetic group include hydrogenases and ferredoxins. Major cellular processes that rely on iron-sulfur cluster proteins include DNA replication and repair, and the sensing of iron as well as oxidative and nitrosative stresses (39). Among those proteins that were dysregulated in WT, we observed that sulfur mobilization pathway proteins SufB, SufC, and SufD were all upregulated. Here, we observed the sulfur mobilization pathway protein SufB to be upregulated in SL samples, with SufC and SufD each also showing a trend toward upregulation, while SufD was identified as significantly dysregulated when considering the combined SL datasets.

In WT, the iron-dependent repressor IdeR (Ms2750), which regulates iron import proteins, was decreased and Ferrochelatase HemH, which incorporates Fe^2+^ into haem, increased. Ms4560, binding protein for Fe^3+^-siderophore transport, was observed to be upregulated, and ferroxidase Bfr, Ms3564, responsible for oxidizing Fe^2+^ to Fe^3+^ was decreased. Of the aforementioned proteins, only HemH was seen to be dysregulated in our SL data, in the combined dataset for these samples, despite all, excepting Bfr, having been quantitated in the SL datasets. We did observe the upregulation of iron import permease Ms6062 and Ferredoxin sulfite reductase, Ms4527, where the latter is in contrast to the downregulation observed in WT samples. We also observed siderophore utilisation protein Ms2511 upregulated in both SL and WT samples, but siderophore synthesis Mbt proteins were only dysregulated in WT samples.

Overall, it is thus clear that iron metabolism is disrupted in both WT and SL mutants and it appears that the sulfur mobilization and porphyrin and haem production pathways are upregulated in both strains. It has previously been hypothesised that rifampicin-related oxidative stresses result in the activation of bacterial programs to capture or sequester free iron as either a protection mechanism against reactive oxygen species (ROS) or as a response to perceived microbial competition (24). If this was the case, we would expect such a response to be evident in our SL samples despite their being comparatively insulated from rifampicin's direct effect of transcriptional interference. This is indeed what we observe, albeit with an absence of dysregulation for several iron import- and incorporation-related enzymes, which were previously observed to be dysregulated in WT.

### Mixed Responses

#### Cell Wall Proteins and Fatty Acid Metabolism

We observed that fatty acid metabolism, biosynthesis and degradation, were among those KEGG pathways enriched for dysregulated proteins (see Supplementary Figure 7). A total of 6 proteins were observed to be dysregulated in SL samples, compared to 14 in WT; 2 of these 6 were common between the 2 strains, but were dysregulated in opposite directions in WT and SL. With three enzymes dysregulated in each of the fatty acid biosynthetic and degradation pathways, and these both upregulated and downregulated, it is difficult to state with confidence the direction in which these pathways as a whole are dysregulated. When we combined our two SL datasets, we observed increases in fatty acid biosynthesis and degradation enzymes (see Supplementary Figure 8), contrary to WT where fatty acid biosynthesis and degradation appeared to be, respectively, increased and decreased.

Phospho-N-acetylmuramoyl-pentapeptide-transferase (MraY) was strongly downregulated in our SL data and, while not achieving significance, showed a similar trend in WT studies (24, 25). In contrast, none of the cell wall synthesis Mur proteins were significantly dysregulated in our SL data, except for MurE, which was upregulated in the combined SL datasets whereas all of MurA, MurC, MurD, MurE, and MurF were upregulated in WT. Similarly, none of UmaA, CwsA, Ddl, PimB, and FbpA were dysregulated in SL bacteria, all of which were previously dysregulated in WT samples (25).

We observed that translocation protein TatB was downregulated, and the metalloprotease FtsH was increased in both SL and WT. Additionally, while we did not observe the previously downregulated cell division protein FtsW in this study, we did observe downregulation in the related protein FtsZ.

Overall, we observed a mix of conserved and non-conserved responses among cell wall proteins. Several proteins followed the same pattern of dysregulation as seen in WT, and fatty acid metabolism showed a similar dysregulation in synthesis but with opposing dysregulation in degradation. However, other responses were almost entirely absent, such as the previous dysregulation of cell wall synthesis CwsA and the Mur proteins. Thus, it is not clear from our data whether the dysregulation of lipid metabolism and cell wall synthesis is conserved between WT and SL strains; future metabolomic studies will thus be required to examine this in more detail.

#### Proteases and Peptidases

**Figure 7:**
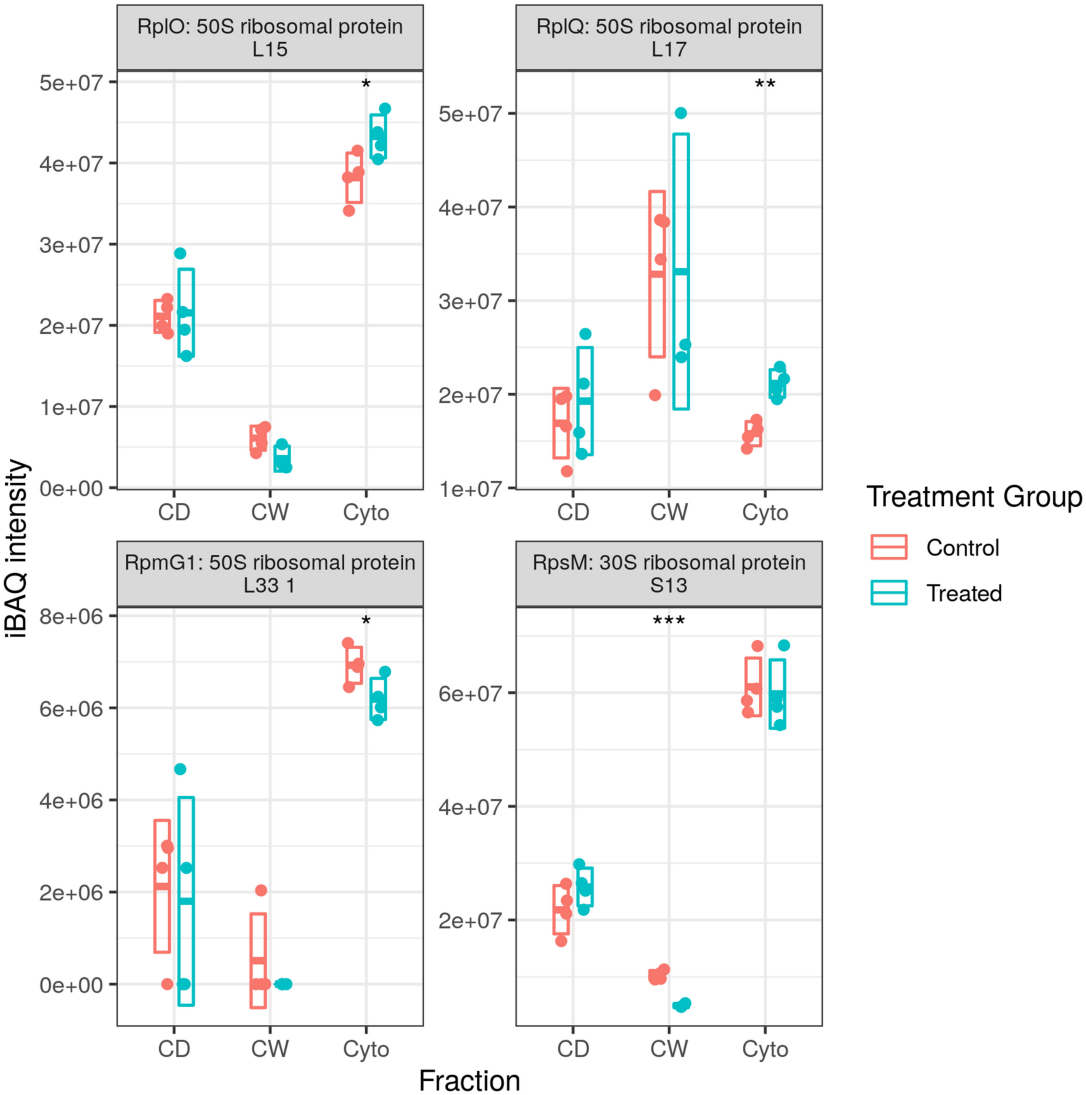
Dysregulated proteases. Examples of protein abundance distributions for several proteases across the different fractions. Rip1 was among the most strongly upregulated proteins in both WT and SL samples. Points indicate individual replicate values and the bars indicate the mean plus/minus SD. Blue indicates control and red indicates rifampicin-treated samples. *, **, and *** indicate *p* < 0.05, *p* < 0.01, and *p* < 0.001 respectively.

We observed numerous proteases and peptidases dysregulated in each of the three proteomic datasets with total numbers of dysregulated proteases and peptidases in our longitudinal WT, cell wall-enriched WT, and this cell wall-enriched SL bacteria study of 17, 13, and 6, respectively. However, while WT studies identified more proteases to be downregulated rather than upregulated, here we identified five of six to be increased upon rifampicin treatment. The only peptidase downregulated was Chymotrypsin Ms5486, an ortholog of Mtb PepD, which was downregulated in the later time points of our longitudinal WT study (24) and was again observed to be downregulated here in both cell debris and cell wall fractions (Figure 7). Conversely, proteasome-associated ATPase Mpa was downregulated in both our longitudinal WT study (time points two and three) and the cell wall-enriched WT study (in both cytosolic and cell debris fractions), while here in our SL study we observed this to be increased in the cytosolic fraction only.

Rip1 was one of the most strongly upregulated proteins in the final time points of the longitudinal study on WT. Here, it was again one of the most strongly upregulated proteins (nearly 6-fold). Rip1 is an intramembrane site-2 protease; named for regulated intramembrane proteolysis, this intramembrane protease triggers a proteolytic cascade upon sensing extracellular stresses, such as oxidative stress, and cleaves membrane bound anti-sigma/sigma factor complexes and penicillin binding protein B, releasing them into the cytoplasm with the aid of an as yet unidentified site-1 protease (40, 41). The anti-sigma factors are fully degraded in the cytoplasm, thereby releasing sigma factors SigK, SigL, and SigM. *M. smegmatis* appears to have twice as many sigma factors encoded in its genome as Mtb yet has no orthologues annotated for SigK or its anti-sigma factor RskA (42). We did not observe the orthologues for SigL or SigM, nor their anti-sigma factors, except for anti-SigL, which was not dysregulated. Nevertheless, taking the union across both studies on WT and this study on SL bacteria, we observed a total of eight distinct sigma factors dysregulated—SigF, MysB, RpoD, SigD, RsfB, Ms2752, Ms0219, and SigH—with five upregulated. This suggests that, even in the absence of a direct effect of rifampicin on RNAP activity in the SL mutant, transcriptional effects may still manifest downstream through the dysregulation of sigma factors.

### Contradictory and Missing Responses

#### Rifampicin-Associated Downregulation of the Hydrogenase Complex Is Not Conserved in Drug-Resistant Bacteria

The *Mycobacterium* genus is highly prevalent in soil, an environment where conditions can rapidly switch from aerobic to anaerobic (43). As such, mycobacteria, despite being classed as obligate aerobes, are able to rapidly adapt to hypoxic conditions, with this metabolic transition controlled by the DosR (DevR, Ms5244) regulon. This genus-wide metabolic plasticity has doubtlessly helped Mtb in developing the capacity to survive in rapidly changing environments as it passes from the moist human lung to comparatively desiccated, highly oxygenated air and then again into a new host, and hypoxic granulomas. In *M. smegmatis*, this adaptive capacity includes hydrogenase-enabled fermentation, with corresponding oxidation when aerobic conditions are restored (43).

In our previous longitudinal study on WT, we observed that hydrogenase maturation and chaperone proteins HypB, HypD, HypE, and HypF were all downregulated in the later time points. In the subsequent cell wall-enrichment study for WT, it was again observed that HypB, HypC, HypD, and HypE were all downregulated in the cytosolic fraction, while HypE was additionally increased in the cell debris fraction. In both WT studies, the two hydrogenase Hyd1 subunits, HybA (Ms2262) and HybC (Ms2263) (43), were strongly (~2-fold) downregulated. The additional hydrogenase maturation protease Ms2264 was also downregulated across these studies. In contrast, in the drug-resistant SL strain, there was no significant difference in the abundance of any of the Hyp or Hyb proteins, nor any other dysregulated hydrogenase maturation proteases, leading us to conclude that this response is not conserved in SL bacteria treated with sublethal rifampicin doses.

#### Dysregulation of Virulence Proteins and Two-Component Systems Is Not Conserved in SL Bacteria

It was previously observed that the important dormancy response regulator DevR, Ms5244, was downregulated in the later time points of the longitudinal study on WT (24). Upon closer inspection, in the fractionated WT dataset (25), it was observed that DevR was decreased in the cytosolic fraction and increased in the cellular debris fraction, possibly indicating increased partitioning of the protein to a DNA-bound state. Here, in SL bacteria, DevR was not dysregulated in either of these fractions, but instead was found to be upregulated in the cell wall fraction. We previously observed that all of the 6 additional DevR regulon proteins dysregulated at this time point (10 in total) in our longitudinal study were decreased in abundance, and 8 of 9 dysregulated in our WT cell wall-enrichment study were downregulated. In our SL data, we observed three DevR regulon proteins dysregulated with no clear overall direction (two up and one down). Thus, it appears that the DevR response was neither strongly induced nor suppressed in SL bacteria upon rifampicin exposure, indicating that previously observed dysregulation served a specific role in the WT bacterial response to rifampicin-induced cell stress. Indeed, this downregulation of the DevR regulon may be linked with the downregulation of the hydrogenase proteins as discussed earlier.

We observed the two-component response regulator PhoP, as well as its cognate histidine sensor kinase PhoR (Ms5870), to be upregulated in SL bacteria as opposed to the downregulation observed in WT. Additionally, none of the companions of PhoP, namely, MtrB (as well as MtrA), PrrB (as well as PrrA), and RegX3 (as well as SenX3)—all downregulated in WT along with PhoP and forming a protein–protein interaction network, with MtrB at the hub—were observed to be dysregulated in this SL data. Further, only 3 two-component system members were dysregulated in SL bacteria, in comparison with 22 in our longitudinal study (9 at this time point), and 22 in our WT cell wall-enrichment study. This implies that two-component systems, whose KEGG pathway includes DevR and the hydrogenase Hyb proteins, were not strongly dysregulated by the presence of rifampicin alone, but rather were dysregulated in response to RpoB-dependent cellular stresses.

## Discussion

### Lack of Conservation in Drug-Resistant Mutant Identifies Responses Triggered by Rifampicin Binding to RpoB

Mycobacteria treated with rifampicin are exposed to multiple potential causes for proteome dysregulation. Firstly, the direct effect of rifampicin on the transcriptional machinery of drug-susceptible bacteria may reduce transcript concentrations, and thus potentially perturb the constitution of the transcriptome, with some transcripts dropping below minimum concentration thresholds necessary to maintain function. Secondly, consequent cellular stress may result in initiation by the cell of a stress response program. Thirdly, rifampicin may induce ROS or other generic stressors, which can engender proteomic responses (including maladaptive ones) (44). Finally, before any of the other potential response instigators, rifampicin may be detected and recognised as a signaling molecule in its own right (13). In considering the effects of rifampicin exposure on the rifampicin-resistant S531L drug-resistant mutant, we eliminate the first two possible causes of a proteomic response, and so can determine that those responses observed in WT which are conserved in SL bacteria are caused either by rifampicin's potential action as a signaling molecule, or in response to some other RpoB-independent effect of drug presence, such as potential oxidative stress or non-specific protein interactions (45). The corollary of this is that those responses observed in WT which are not conserved in SL mutant bacteria are likely dysregulated as part of an adaptive response to stresses induced *via* rifampicin's specific activity on the RNAP (24).

Our study thus reveals that some responses previously assumed to be related to oxidative stress were in fact consequent to the effect of rifampicin on the bacteria *via* transcriptional inhibition. One such response is the downregulation of hydrogenase complex proteins that was observed in WT but was nearly completely absent in SL mycobacteria. Given the evidence for conserved dysregulation in the haem biosynthetic pathway, and the dysregulation of iron and sulfur metabolising enzymes, albeit, with evidence weaker than that observed in WT, it was surprising to see downregulation of the hydrogenase proteins not be conserved. Related to this, “Two-component System” regulators, one of the most enriched KEGG pathways in WT studies, was only the 40th most enriched term here with far fewer dysregulated proteins than observed in our WT studies (24, 25). Another example is provided by virulence-associated PknG, which was one of the most strongly downregulated (>3-fold) proteins in WT studies, being downregulated in all the three cellular fractions (25), whereas in this SL data, we failed to identify any significant difference in PknG protein abundance. In all the three cases, we can conclude that these responses were dependent on the direct action of rifampicin on RpoB and part of the bacterial response to rifampicin-induced stress. The functional consequence of the observed downregulation of PknG in promoting resistance to rifampicin (46) remains to be determined, the caveat being that from the present data alone we are unable to determine whether PknG genuinely decreases in abundance following rifampicin treatment in WT bacteria, or whether the apparent decrease of PknG in the cell wall fractions is in fact the result of increased secretion. Further targeted proteomic studies on culture supernatant are needed to resolve this ambiguity and will be reported elsewhere.

Overall, it appears that many important two-component systems, such as MtrAB, are dysregulated in WT in consequence of rifampicin-induced, RpoB-dependent stress and not the presence of rifampicin as a direct signaling molecule.

### Decreased Abundance of ABC Transporters Is a Specific Response to Sublethal Rifampicin in Drug Sensitive and Resistant *M. smegmatis*

A striking result from the present study is the conserved downregulation of ABC transporters, in accordance with our previous observations in both longitudinal and cell wall-enriched WT bacteria. That such a response is conserved in a drug-resistant strain, which experiences no phenotypically observable stress, suggests that this dysregulation is prompted by an upstream molecular signaling network. Given the strong, consistent upregulation of the intramembrane protease Rip1, and the strong, consistent downregulation of ABC transporters, we considered whether these observations might plausibly be connected. However, while it is known that, in *Bacillus subtilis*, the anti-sigma factor of the σ^*W*^ regulon, RsiW, is controlled by regulated intramembrane proteolysis that is itself dependent on an ABC transporter (47), we are not aware of any prior literature that might causally connect the upregulation of Rip1 with the downregulation of ABC transporters. This possibility thus warrants a further metabolic labeling-based protein turnover study in due course.

To determine whether the downregulation of ABC transporters could be the result of a response to oxidative stress, we re-analysed the data from a previous study by Ganief et al. (48) using the same bioinformatic analysis script employed in previous WT studies and in this study. From our re-analysis, Ganief et al. (48) observed greater ABC transporter dysregulation upon bacterial exposure to nitric oxide stress than upon H_2_O_2_ exposure, with 8 of 12 ABC transporters decreased upon NO stress. Only three of the seven ABC transporters in common between our WT studies and those observed by Ganief et al. (48) were dysregulated in the same direction. In particular, we note that Ms3250, downregulated in both WT and SL studies in response to rifampicin, was likewise downregulated in response to NO, whereas Ms5897, decreased in both WT and SL data in response to rifampicin, was increased upon NO exposure. While these observations by Ganief et al. (48) were made with different time points to those employed in this study, it seems that oxidative stress can induce downregulation in ABC transporters—with this more evident with reactive nitrogen intermediates than with ROS—but that the specific overlap of ABC transporters with that observed with rifampicin exposure is low. In contrast, we observe good overlap between rifampicin-treated SL and WT results. This suggests that there is a pattern of downregulated ABC transporters and related proteins specific to rifampicin exposure, which is conserved across drug resistance status and is distinct from that observed in response to oxidative stress.

The conserved downregulation of ABC transporters in response to rifampicin presence could impact bacterial permeability to drug—both through impeding active transport across the cell envelope and by altering the protein composition of the envelope—and so potentially serve as a protection mechanism, rendering mycobacteria more resistant to other drugs in addition to rifampicin. Such an effect has been reported previously, with the upregulation of efflux pumps leading to resistance against multiple drugs (49–51) and with rifampicin-induced ofloxacin resistance (52). If this likely ancestral response to rifampicin exposure, involving the rapid downregulation of ABC transporters, is conserved in *Mtb—*and if it is indeed protective, as hypothesised here—it could in principle be targeted for disruption, with the goal of increasing intracellular drug concentrations of existing anti-TB drugs and counteracting cross-resistance.

### Upregulation of Transcriptional and Translational Machinery in Response to Rifampicin Occur Independently of Each Other

It is tempting to speculate that the conserved upregulation of ribosomal protein abundance in response to rifampicin is evidence for specific recognition of rifampicin in mycobacteria, rather than of a general stress response. However, this seems plausible only in the scenario that transcriptional machinery was equally influenced as this is the primary target of rifampicin. While it may yet be that an equal response with respect to transcriptional machinery does occur, the proteomic data presented here does not support that possibility. We therefore propose an alternative, in which these two major biosynthetic activities are uncoupled in mycobacteria. Fukuda et al. showed that in *E. coli* the *rpoBC* operon could be regulated separately to ribosomal machinery when treated with rifampicin and that this decoupling could be attributed to the binding of rifampicin to the RNAP interfering not only with transcriptional activity but also with the autogenous repression by the RNAP of its own transcription (53–55). By extrapolation, we thus propose that in WT mycobacteria, rifampicin can induce increased RNAP expression, but in S531L mutants, the very mechanism that provides the protection from transcriptional inhibition also prevents the induction of RNAP. In other words, mycobacterial upregulation of transcriptional machinery seems likely to be a product of the direct binding of rifampicin to the RNAP and not of the recognition of its presence *via* alternative sensing mechanisms.

In terms of the observed, uncoupled upregulation of translational machinery, we specifically noted that many downregulated ABC transporters are essential for the uptake of amino acids as well as of precursors thereof. Thus, it may be that the upregulation of translational machinery follows the same logic for the upregulation of aminoacyl-tRNA synthesis: reduced availability of amino acids (and hence charged tRNA substrate) for translation caused by the reduced cellular import of extracellular nutrients through a decreased abundance of ABC transporters necessitates the upregulation of ribosomal machinery to improve translation rates.

### Limitations of This Study

A limitation of the present study is that passive diffusion of rifampicin across the cell envelope may be affected by changes in the lipid or protein composition of the cell envelope, and we cannot discount the possibility that WT and SL mutants experience different intracellular rifampicin concentrations as a result. Furthermore, as one of the early proteomic events we observe in both WT and SL mutants is the rapid downregulation of numerous ABC transporters upon exposure to sublethal rifampicin, it seems probable that there is a further dynamic downstream effect on drug permeability that is not necessarily identical in WT and SL mutants. However, while this is undoubtedly an interesting question, it is important to reiterate that this study was not designed to answer questions surrounding differences in intrinsic rifampicin permeability, or in dynamic changes in permeability, or in intracellular rifampicin concentrations following initial rifampicin exposure. Instead, this study was designed primarily to identify whether any of the specific, early rifampicin-dependent proteomic changes were conserved between WT and SL mutants—which were indeed observed. A further limitation of this study is that the functional consequences of the observed dysregulation of the *M. smegmatis* proteome in the presence of sublethal rifampicin remain to be validated. As is typical with discovery proteomics experiments, many new hypotheses have been generated in this study which warrant further, detailed mechanistic investigation. However, the primary objective of this study was to determine which of the proteomic changes observed in the WT were conserved in the SL mutant and was not to explore the downstream functional consequences of the changes observed in the WT but not in the SL mutant.

## Conclusion

We treated rifampicin-resistant S531L RpoB mutant *M. smegmatis* with a sublethal concentration of rifampicin and compared the induced, adaptive proteomic responses with previous studies on WT strains to determine which responses were conserved across drug resistance status and which responses were specifically associated with drug activity. We observed conservation of the downregulation of ABC transporters and the upregulation of translational machinery, which are plausibly linked mechanistically through the restriction of amino acid import and biosynthesis. Evidence for upregulated transcriptional machinery in the SL strain was notably lacking, indicating that this is regulated separately from translational machinery by direct rifampicin-RNAP binding. In addition, we observed mixed evidence for the conserved dysregulation of the porphyrin and vitamin B12 pathway and iron metabolism, as well as a notable absence of dysregulation of any hydrogenase subunits or related proteins in the SL strain. Taken together, our data show that rifampicin engenders two distinct patterns of proteomic dysregulation: one associated with cellular stress caused by rifampicin binding to RpoB and the other an RpoB-independent pattern, which in turn suggests that rifampicin may be specifically recognised as a signaling molecule by mycobacteria through an as yet unidentified upstream sensor. Further phosphoproteomic work to identify this putative upstream sensor is underway and will be reported elsewhere.

Collectively, our findings demonstrate that a number of key phenotypic adaption mechanisms in *M. smegmatis* in the presence of sublethal rifampicin continue to occur in a rifampicin monoresistant strain. Most notably, the rapid downregulation of multiple ABC transporters, even in a rifampicin mono-resistant strain, points toward a rifampicin-dependent, but RpoB-independent pathway with clear implications for the subsequent acquisition of multidrug resistance, by promoting increased phenotypic resistance to the cellular import of other antimycobacterial drugs. Further work to explore this possibility, as well as determine whether similar RpoB-independent phenotypic adaption occurs in *M. tuberculosis*, is now underway.

## Data Availability Statement

The data presented in the study are deposited in the Proteome Identifications (PRIDE) database repository, accession number PXD027855 (https://www.ebi.ac.uk/pride/archive/projects/PXD027855).

## Author Contributions

JB and NS contributed to conception and design of the study. TG and NG contributed to the LC-MS/MS analysis. DW and AK generated the mutant strain. AG performed the lab work, data processing, and statistical analysis. AG and JB wrote and edited the manuscript. All authors contributed to the article and approved the submitted version.

## Funding

This work was based on research supported by the National Research Foundation (NRF) of South Africa (Grant Number 64760).

## Conflict of Interest

The authors declare that the research was conducted in the absence of any commercial or financial relationships that could be construed as a potential conflict of interest.

## Publisher's Note

All claims expressed in this article are solely those of the authors and do not necessarily represent those of their affiliated organizations, or those of the publisher, the editors and the reviewers. Any product that may be evaluated in this article, or claim that may be made by its manufacturer, is not guaranteed or endorsed by the publisher.
