# Supplementary material for: Cell Wall Proteomics Reveal Phenotypic Adaption of Drug-Resistant Mycobacterium smegmatis to Subinhibitory Rifampicin Exposure
Source: Front Med (Lausanne). 2021 Oct 5;8:723667. doi: 10.3389/fmed.2021.723667 (PMC8525676; doi:10.3389/fmed.2021.723667)
Supplement: Supplementary file 2 [file Data_Sheet_2.docx]

Supplementary Material

# Supplementary Figures

## Cell Wall Enrichment Schema


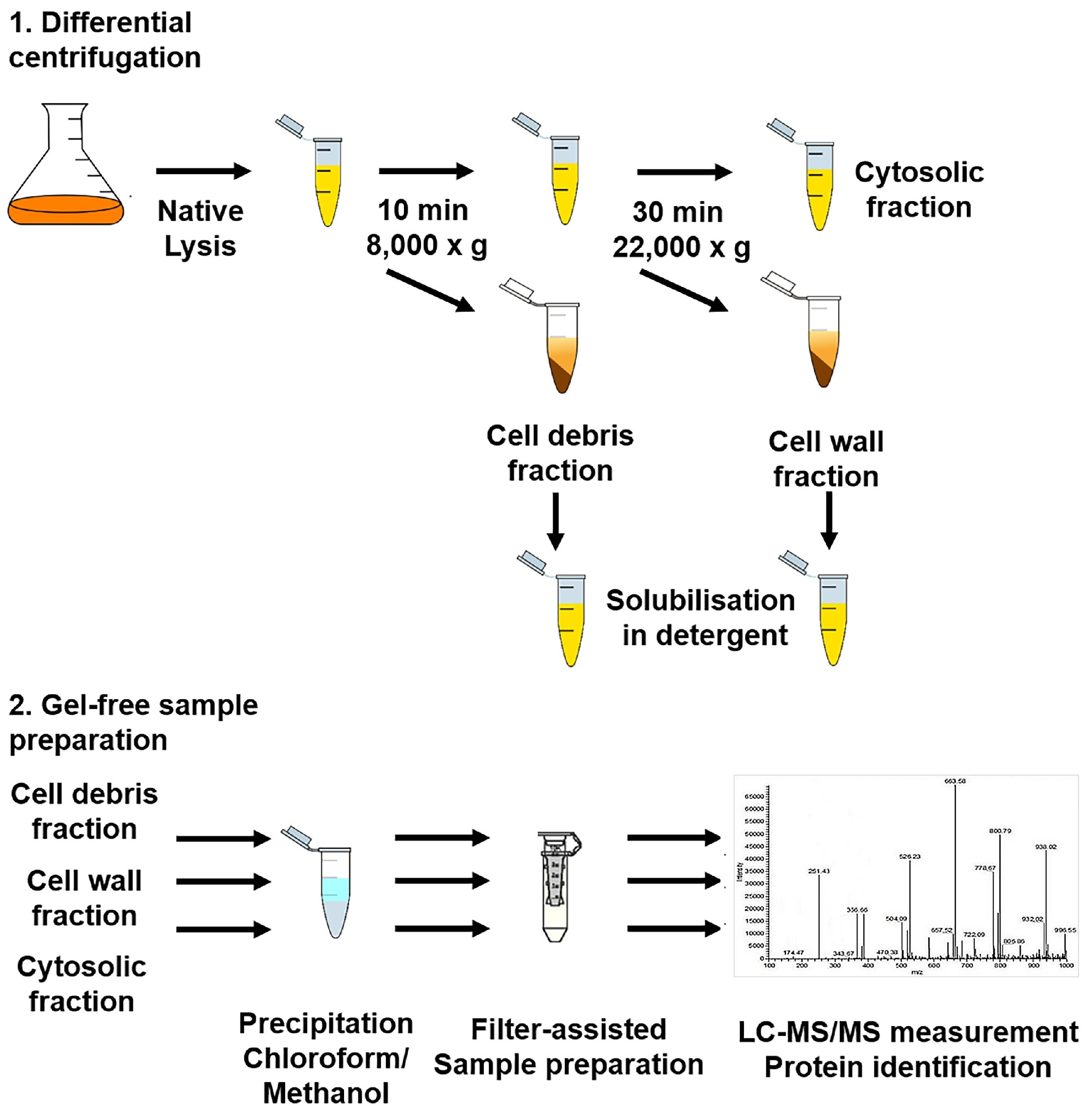


Figure S1: **Schema of Cell Wall Protein Enrichment and Preparation of Resulting Cellular Fractions**. Cellular debris was precipitated from lysate via centrifugation at 8,000 RCF, washed and boiled in detergent. The cell wall fraction was precipitated from the prior supernatant by centrifugation at 22,000 RCF, washed and solubilised in detergent. The resulting supernatant was the cytosolic fraction. Protein from each fraction was precipitated by chloroform and methanol and prepared for analysis by FASP. Figure adapted from Hermann et al (55).

## Data Quality of Supplemental Dataset


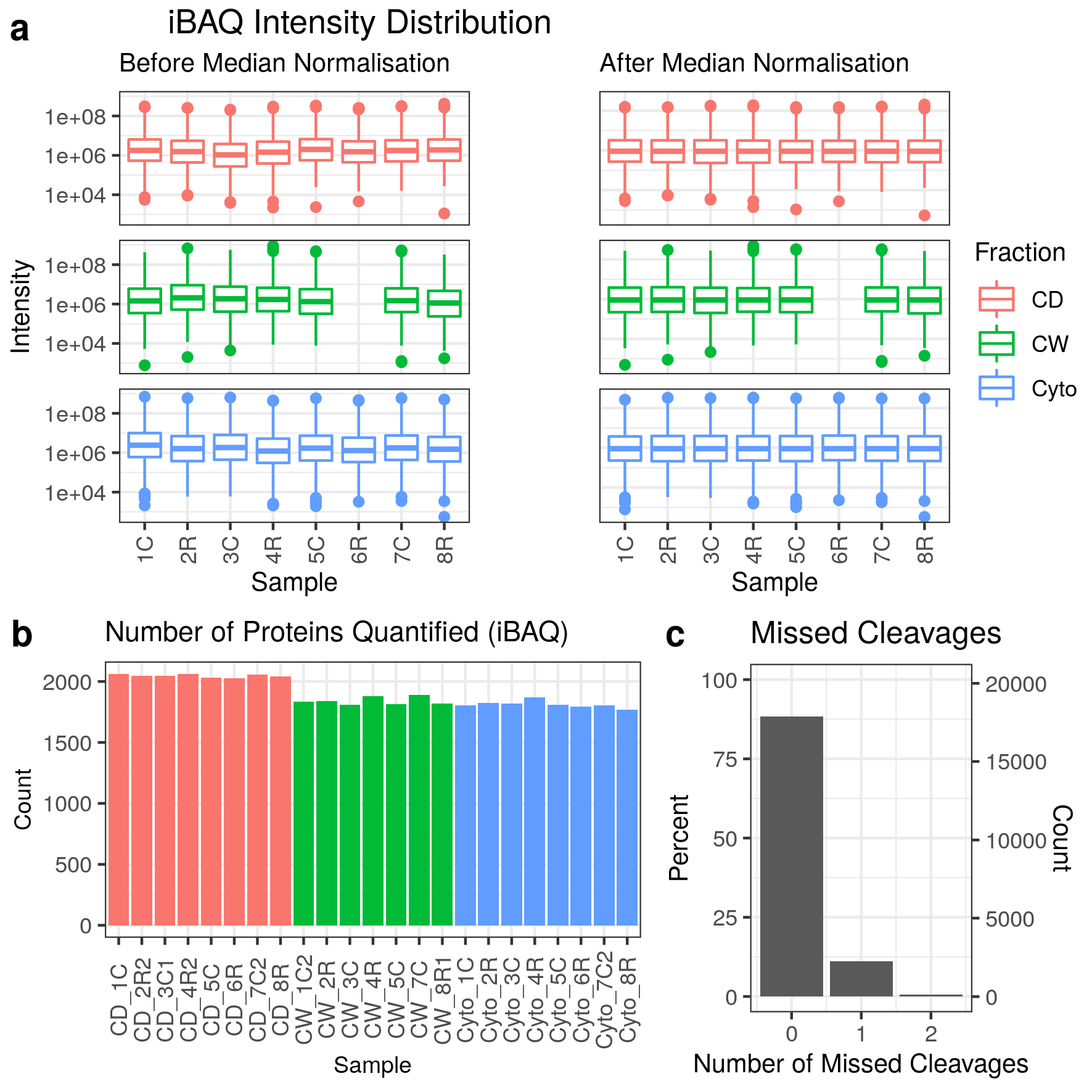


Figure S2: **Data Quality for Supplementary Dataset**. **a)** Distribution of iBAQ protein quantifications by sample both before and after median normalisation. **b)** Number of quantified proteins by sample. Colour scheme for panels a and b is red for cell debris, green for cell wall and blue for cytosolic fractions. **c)** Frequency of number of missed trypsin cleavage sites in identified peptides revealing nearly 90% of identified peptides showed no missed cleavage sites.

## Principal Component Analyses


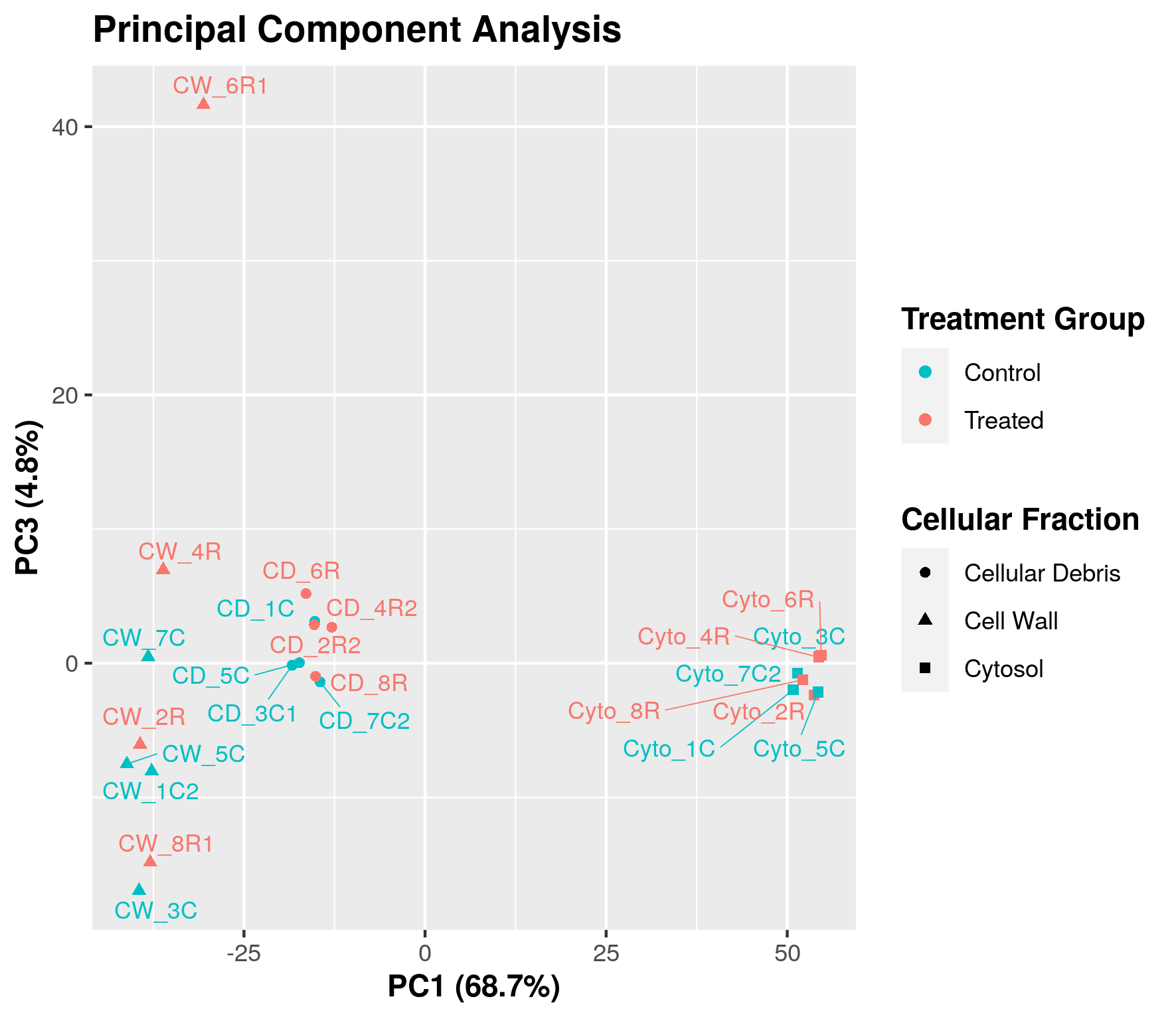


Figure S3: **Principal Component Analysis**. A principal component analysis identifying the cell wall fraction of sample 6R as an outlier. Rifampicin treated samples are shown in red and control samples in blue. Cellular debris, cell wall and cytosolic fractions are indicated by dots, triangles and squares respectively. Principal components one and three account for 68.7% and 4.8% of total variation respectively.


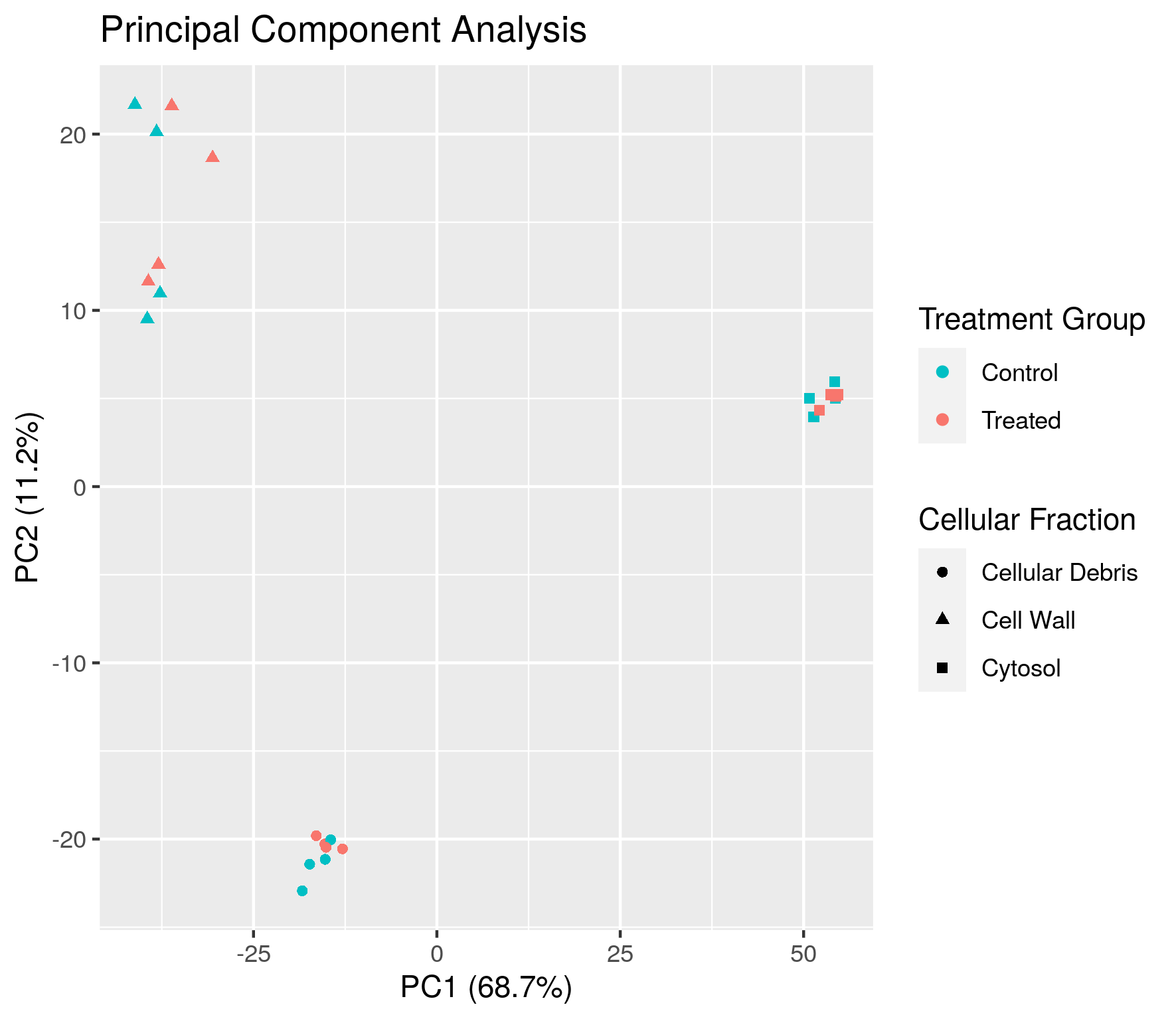


Figure S4: **Principal Component Analysis**. A principal component analysis showing strong separation of samples according to cellular fraction. Rifampicin treated samples are shown in red and control samples in blue. Cellular debris, cell wall and cytosolic fractions are indicated by dots, triangles and squares respectively. Principal components one and two account for 68.7% and 11.2% of total variation respectively.

## Ribosomal Proteins


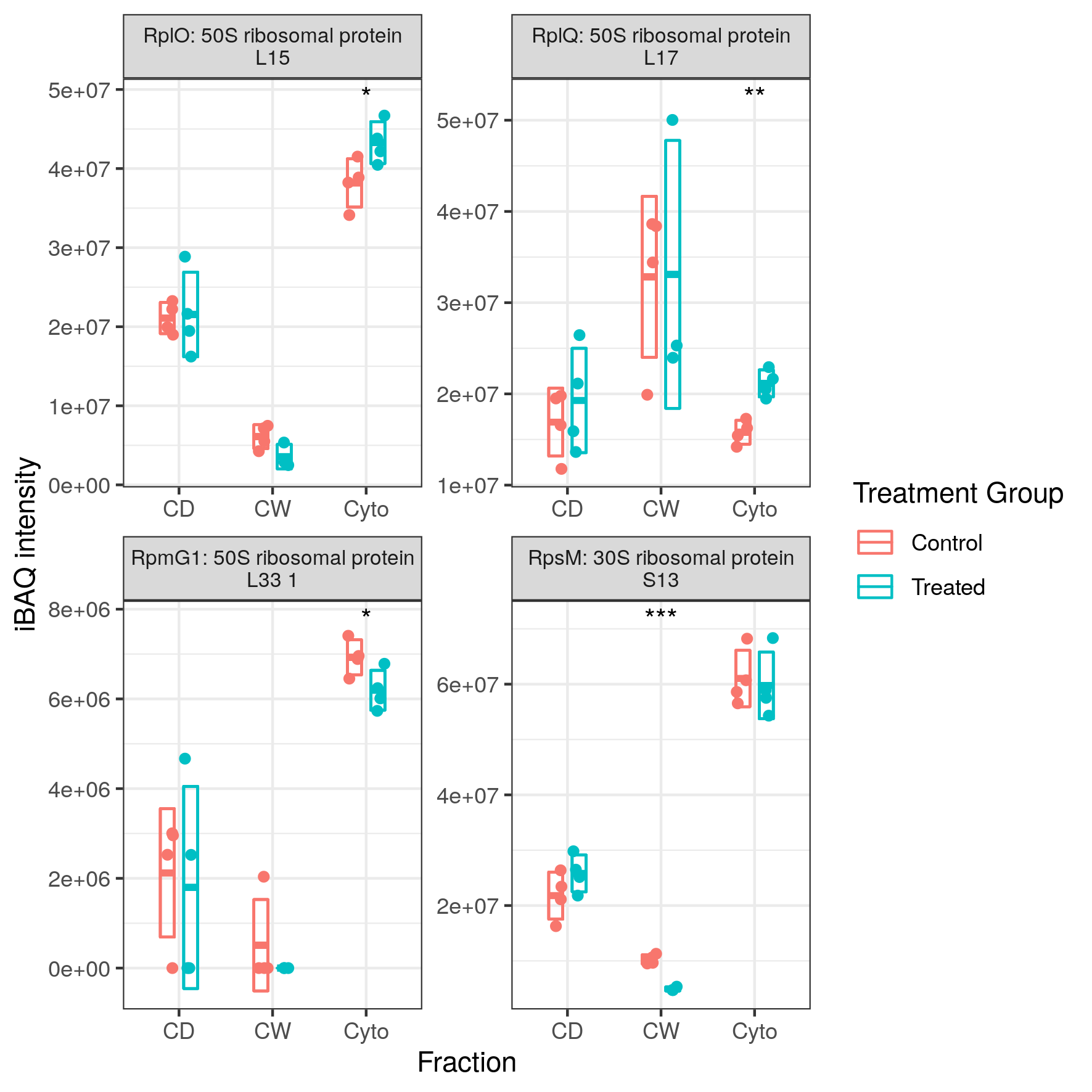


Figure S5: **Expression Profiles for Several Ribosomal Subunit Proteins**. Ribosomal proteins were both up- and downregulated with RplQ and RplO upregulated and RpsM and RpmG1 downregulated in at least one fraction each. Points indicate individual replicate values measured, bars indicate mean expression with error bars indicating standard deviation. Control samples shown in blue and rifampicin treated in red.

## Iron and Vitamin B12 Metabolism


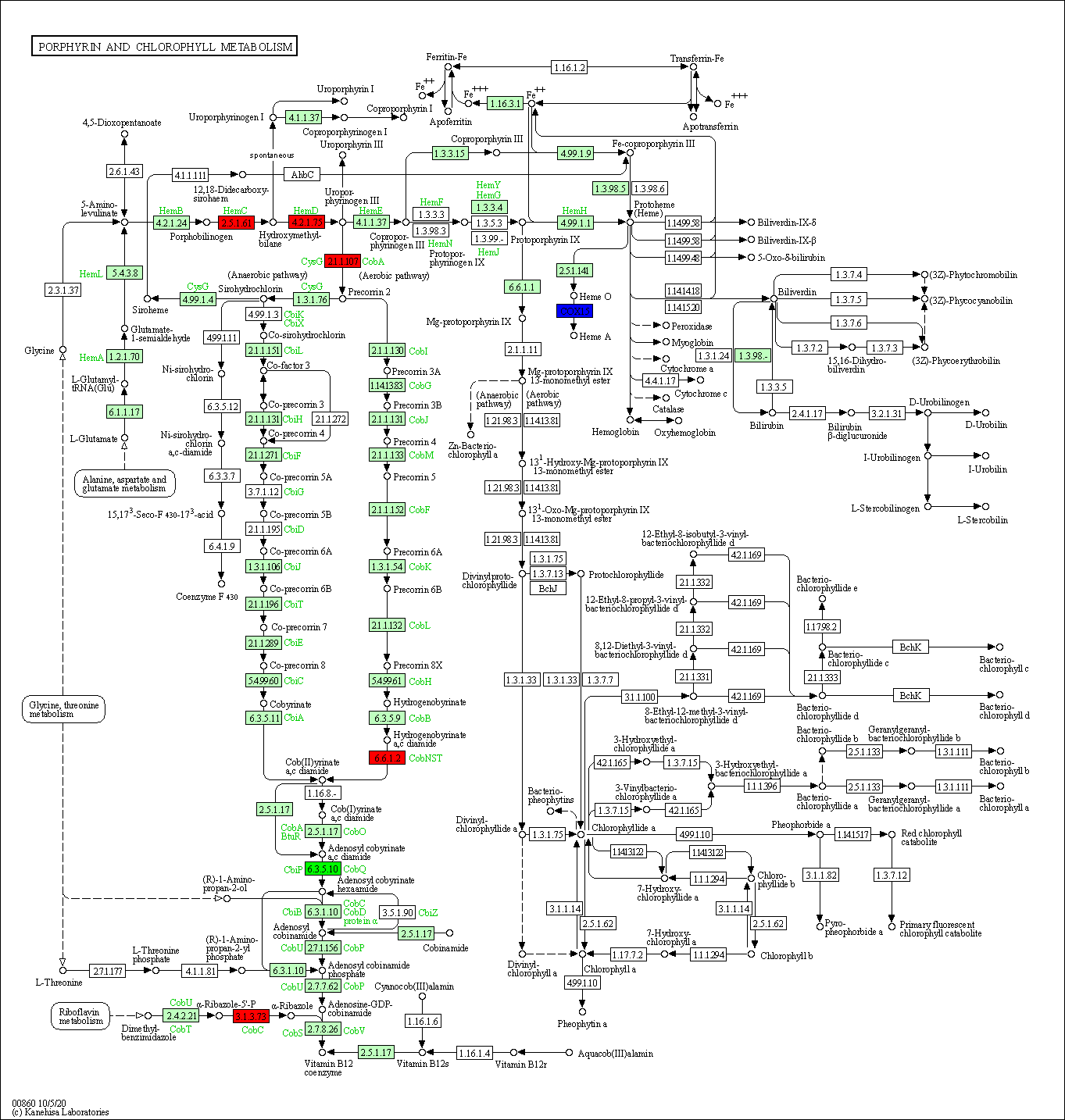


Figure S6: **Dysregulation of Porphyrin and Vitamin B12 Synthesis Pathway in Drug Resistant Mutant**. Blue and red coloured boxes indicate the relevant protein was respectively down- or upregulated in one or more fractions. Green indicates protein was observed to be both up- and downregulated in different cellular fractions.

## GO Term and KEGG Pathway Enrichment


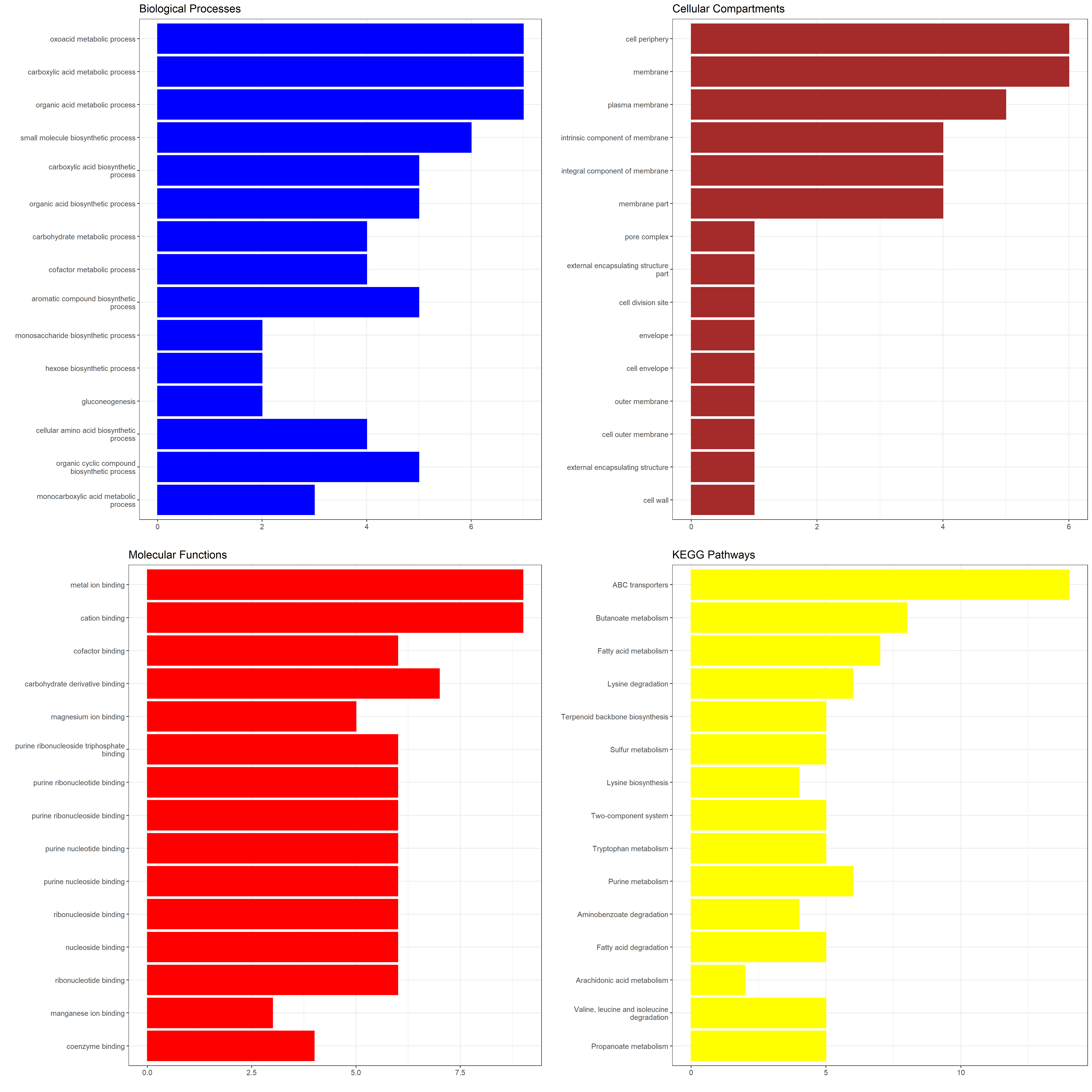


Figure S7: **GO Term Enrichment of Proteins Dysregulated in Drug Resistant SL Mycobacteria**. Top 15 gene ontology terms with p<0.05 after multiple testing correction. Terms ordered by ascending p-value for enrichment (greatest enrichment at the top).

## Fatty Acid Metabolism


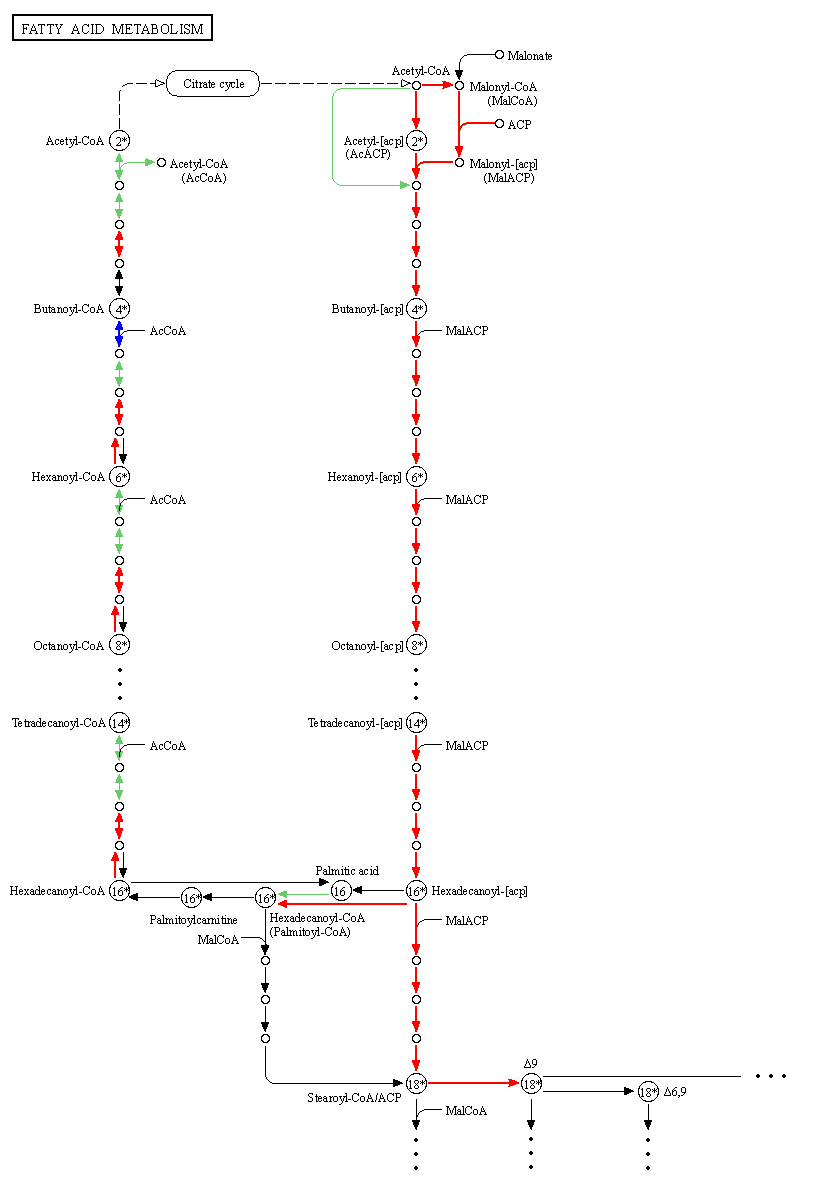


Figure S8: **KEGG Pathway for Lipid Metabolism**. The combined SL dataset was used to construct this plot. Arrows indicate reactions catalysed by enzymes and circles indicate product intermediates. Red arrows indicate enzymes are increased and blue decreased in samples treated with rifampicin as compared with control samples. Overall we see that both fatty acid biosynthesis and degradation show evidence for being upregulated. Please note that this image represents the generic pathway and some steps have no prokaryotic enzyme mapping, such as the synthesis of Palmitoylcarnitine.
